# Supplementary material for: Modeling reciprocal effects in medical research: Critical discussion on the current practices and potential alternative models
Source: PLoS One. 2019 Sep 27;14(9):e0209133. doi: 10.1371/journal.pone.0209133 (PMC6764673; doi:10.1371/journal.pone.0209133)
Supplement: S1 File — (PDF) [file pone.0209133.s001.pdf]

Supplemental Online Materials

1. Lavaan codes for the CLPM, the RI-CLPM and the STARTS model·············· p.2

2. Simulation codes for frequency of improper solutions································ p.5

3. Simulation codes for statistical properties of estimates····························· p.11

4. Table A: full references for retained papers·· p.17

5. Table B: Marginal means of [standardized] estimates/biases of cross-lagged parameters at each model··· p.40

1. Lavaan codes for the CLPM, the RI-CLPM and the STARTS model

#Lavaan code for the CLPM

#CLPM(T=4)

```
CLPMT4 <- '
x1~mux1*1; y1~muy1*1; x2~mux2*1; y2~muy2*1; x3~mux3*1; y3~muy3*1; x4~mux4*1; y4~muy4*1;
FFx1~0*1;FFy1~0*1; FFx1~~phix*FFx1; FFy1~~phiy*FFy1; FFx1~~phixy*FFy1;
FFy2~ betay*FFy1+gammay*FFx1; FFx2~ betax*FFx1+gammax*FFy1
FFy3~ betay*FFy2+gammay*FFx2; FFx3~ betax*FFx2+gammax*FFy2
FFy4~ betay*FFy3+gammay*FFx3; FFx4~ betax*FFx3+gammax*FFy3
FFx2~~Omegax*FFx2; FFx3~~Omegax*FFx3; FFx4~~Omegax*FFx4;
FFy2~~Omegay*FFy2; FFy3~~Omegay*FFy3; FFy4~~Omegay*FFy4;
FFx2~~Omegaxy*FFy2; FFx3~~Omegaxy*FFy3; FFx4~~Omegaxy*FFy4;
FFy1 =~ 1*y1; FFy2 =~ 1*y2; FFy3 =~ 1*y3; FFy4 =~ 1*y4
FFx1 =~ 1*x1; FFx2 =~ 1*x2; FFx3 =~ 1*x3; FFx4 =~ 1*x4

'
```

#CLPM(T=6)

```
CLPMT6 <- '
x1~mux1*1; y1~muy1*1; x2~mux2*1; y2~muy2*1; x3~mux3*1; y3~muy3*1; x4~mux4*1; y4~muy4*1;
x5~mux5*1; y5~muy5*1; x6~mux6*1; y6~muy6*1
FFx1~0*1;FFy1~0*1; FFx1~~phix*FFx1; FFy1~~phiy*FFy1; FFx1~~phixy*FFy1;
FFy2~ betay*FFy1+gammay*FFx1; FFx2~ betax*FFx1+gammax*FFy1
FFy3~ betay*FFy2+gammay*FFx2; FFx3~ betax*FFx2+gammax*FFy2
FFy4~ betay*FFy3+gammay*FFx3; FFx4~ betax*FFx3+gammax*FFy3
FFy5~ betay*FFy4+gammay*FFx4; FFx5~ betax*FFx4+gammax*FFy4
FFy6~ betay*FFy5+gammay*FFx5; FFx6~ betax*FFx5+gammax*FFy5
FFx2~~Omegax*FFx2; FFx3~~Omegax*FFx3; FFx4~~Omegax*FFx4; FFx5~~Omegax*FFx5; FFx6~~Omegax*FFx6
FFy2~~Omegay*FFy2; FFy3~~Omegay*FFy3; FFy4~~Omegay*FFy4; FFy5~~Omegay*FFy5; FFy6~~Omegay*FFy6
FFx2~~Omegaxy*FFy2; FFx3~~Omegaxy*FFy3; FFx4~~Omegaxy*FFy4; FFx5~~Omegaxy*FFy5; FFx6~~Omegaxy*FFy6
FFy1 =~ 1*y1; FFy2 =~ 1*y2; FFy3 =~ 1*y3; FFy4 =~ 1*y4; FFy5 =~ 1*y5; FFy6 =~ 1*y6
FFx1 =~ 1*x1; FFx2 =~ 1*x2; FFx3 =~ 1*x3; FFx4 =~ 1*x4; FFx5 =~ 1*x5; FFx6 =~ 1*x6
'
```

#CLPM(T=8)

```
CLPMT8 <- '
x1~mux1*1; y1~muy1*1; x2~mux2*1; y2~muy2*1; x3~mux3*1; y3~muy3*1; x4~mux4*1; y4~muy4*1;
x5~mux5*1; y5~muy5*1; x6~mux6*1; y6~muy6*1; x7~mux7*1; y7~muy7*1; x8~mux8*1; y8~muy8*1;
FFx1~0*1;FFy1~0*1; FFx1~~phix*FFx1; FFy1~~phiy*FFy1; FFx1~~phixy*FFy1;
FFy2~ betay*FFy1+gammay*FFx1; FFx2~ betax*FFx1+gammax*FFy1
FFy3~ betay*FFy2+gammay*FFx2; FFx3~ betax*FFx2+gammax*FFy2
FFy4~ betay*FFy3+gammay*FFx3; FFx4~ betax*FFx3+gammax*FFy3
FFy5~ betay*FFy4+gammay*FFx4; FFx5~ betax*FFx4+gammax*FFy4
FFy6~ betay*FFy5+gammay*FFx5; FFx6~ betax*FFx5+gammax*FFy5
FFy7~ betay*FFy6+gammay*FFx6; FFx7~ betax*FFx6+gammax*FFy6
FFy8~ betay*FFy7+gammay*FFx7; FFx8~ betax*FFx7+gammax*FFy7
FFx2~~Omegax*FFx2; FFx3~~Omegax*FFx3; FFx4~~Omegax*FFx4; FFx5~~Omegax*FFx5; FFx6~~Omegax*FFx6; FFx7~~Omegax*FFx7; FFx8~~Omegax*FFx8
FFy2~~Omegay*FFy2; FFy3~~Omegay*FFy3; FFy4~~Omegay*FFy4; FFy5~~Omegay*FFy5; FFy6~~Omegay*FFy6; FFy7~~Omegay*FFy7; FFy8~~Omegay*FFy8
FFx2~~Omegaxy*FFy2; FFx3~~Omegaxy*FFy3; FFx4~~Omegaxy*FFy4; FFx5~~Omegaxy*FFy5; FFx6~~Omegaxy*FFy6; FFx7~~Omegaxy*FFy7;
FFx8~~Omegaxy*FFy8
FFy1 =~ 1*y1; FFy2 =~ 1*y2; FFy3 =~ 1*y3; FFy4 =~ 1*y4; FFy5 =~ 1*y5; FFy6 =~ 1*y6; FFy7 =~ 1*y7; FFy8 =~ 1*y8
FFx1 =~ 1*x1; FFx2 =~ 1*x2; FFx3 =~ 1*x3; FFx4 =~ 1*x4; FFx5 =~ 1*x5; FFx6 =~ 1*x6; FFx7 =~ 1*x7; FFx8 =~ 1*x8
'
```

#Lavaan code for the RI-CLPM

```
#RI-CLPM(T=4)
	RICLPMT4 <- '
	x1~mux1*1; y1~muy1*1; x2~mux2*1; y2~muy2*1; x3~mux3*1; y3~muy3*1; x4~mux4*1; y4~muy4*1;
	x1~~0*x1; y1~~0*y1; x2~~0*x2; y2~~0*y2; x3~~0*x3; y3~~0*y3; x4~~0*x4; y4~~0*y4
	Tx~~0*FFx1;Tx~~0*FFy1; Ty~~0*FFx1;Ty~~0*FFy1;
	Tx=~1*x1+1*x2+1*x3+1*x4; Ty=~1*y1+1*y2+1*y3+1*y4
	Tx~0*1; Ty~0*1; Tx~~taux*Tx; Ty~~tauy*Ty; Tx~~tauxy*Ty
	FFx1~0*1;FFy1~0*1; FFx1~~phix*FFx1; FFy1~~phiy*FFy1; FFx1~~phixy*FFy1;
	FFy2~ betay*FFy1+gammay*FFx1; FFx2~ betax*FFx1+gammax*FFy1
	FFy3~ betay*FFy2+gammay*FFx2; FFx3~ betax*FFx2+gammax*FFy2
	FFy4~ betay*FFy3+gammay*FFx3; FFx4~ betax*FFx3+gammax*FFy3
	FFx2~~Omegax*FFx2; FFx3~~Omegax*FFx3; FFx4~~Omegax*FFx4;
	FFy2~~Omegay*FFy2; FFy3~~Omegay*FFy3; FFy4~~Omegay*FFy4;
	FFx2~~Omegaxy*FFy2; FFx3~~Omegaxy*FFy3; FFx4~~Omegaxy*FFy4;
	FFy1 =~ 1*y1; FFy2 =~ 1*y2; FFy3 =~ 1*y3; FFy4 =~ 1*y4
	FFx1 =~ 1*x1; FFx2 =~ 1*x2; FFx3 =~ 1*x3; FFx4 =~ 1*x4

'

#RI-CLPM(T=6)
  RICLPMT6 <- '
	x1~mux1*1; y1~muy1*1; x2~mux2*1; y2~muy2*1; x3~mux3*1; y3~muy3*1; x4~mux4*1; y4~muy4*1;
	x5~mux5*1; y5~muy5*1; x6~mux6*1; y6~muy6*1
	x1~~0*x1; y1~~0*y1; x2~~0*x2; y2~~0*y2; x3~~0*x3; y3~~0*y3; x4~~0*x4; y4~~0*y4; x5~~0*x5; y5~~0*y5; x6~~0*x6;
y6~~0*y6;
	Tx~~0*FFx1;Tx~~0*FFy1; Ty~~0*FFx1;Ty~~0*FFy1;
	Tx=~1*x1+1*x2+1*x3+1*x4+1*x5+1*x6; Ty=~1*y1+1*y2+1*y3+1*y4+1*y5+1*y6
	Tx~0*1; Ty~0*1; Tx~~taux*Tx; Ty~~tauy*Ty; Tx~~tauxy*Ty
	FFx1~0*1;FFy1~0*1; FFx1~~phix*FFx1; FFy1~~phiy*FFy1; FFx1~~phixy*FFy1;
	FFy2~ betay*FFy1+gammay*FFx1; FFx2~ betax*FFx1+gammax*FFy1
	FFy3~ betay*FFy2+gammay*FFx2; FFx3~ betax*FFx2+gammax*FFy2
	FFy4~ betay*FFy3+gammay*FFx3; FFx4~ betax*FFx3+gammax*FFy3
	FFy5~ betay*FFy4+gammay*FFx4; FFx5~ betax*FFx4+gammax*FFy4
	FFy6~ betay*FFy5+gammay*FFx5; FFx6~ betax*FFx5+gammax*FFy5
	FFx2~~Omegax*FFx2; FFx3~~Omegax*FFx3; FFx4~~Omegax*FFx4; FFx5~~Omegax*FFx5; FFx6~~Omegax*FFx6
	FFy2~~Omegay*FFy2; FFy3~~Omegay*FFy3; FFy4~~Omegay*FFy4; FFy5~~Omegay*FFy5; FFy6~~Omegay*FFy6
	FFx2~~Omegaxy*FFy2; FFx3~~Omegaxy*FFy3; FFx4~~Omegaxy*FFy4; FFx5~~Omegaxy*FFy5; FFx6~~Omegaxy*FFy6
	FFy1 =~ 1*y1; FFy2 =~ 1*y2; FFy3 =~ 1*y3; FFy4 =~ 1*y4; FFy5 =~ 1*y5; FFy6 =~ 1*y6
	FFx1 =~ 1*x1; FFx2 =~ 1*x2; FFx3 =~ 1*x3; FFx4 =~ 1*x4; FFx5 =~ 1*x5; FFx6 =~ 1*x6
'

#RI-CLPM(T=8)
  RICLPMT8 <- '
	x1~mux1*1; y1~muy1*1; x2~mux2*1; y2~muy2*1; x3~mux3*1; y3~muy3*1; x4~mux4*1; y4~muy4*1;
	x5~mux5*1; y5~muy5*1; x6~mux6*1; y6~muy6*1; x7~mux7*1; y7~muy7*1; x8~mux8*1; y8~muy8*1;
	x1~~0*x1; y1~~0*y1; x2~~0*x2; y2~~0*y2; x3~~0*x3; y3~~0*y3; x4~~0*x4; y4~~0*y4; x5~~0*x5; y5~~0*y5; x6~~0*x6;
y6~~0*y6; x7~~0*x7; y7~~0*y7; x8~~0*x8; y8~~0*y8
	Tx~~0*FFx1;Tx~~0*FFy1; Ty~~0*FFx1;Ty~~0*FFy1;
	Tx=~1*x1+1*x2+1*x3+1*x4+1*x5+1*x6+1*x7+1*x8; Ty=~1*y1+1*y2+1*y3+1*y4+1*y5+1*y6+1*y7+1*y8
	Tx~0*1; Ty~0*1; Tx~~taux*Tx; Ty~~tauy*Ty; Tx~~tauxy*Ty
	FFx1~0*1;FFy1~0*1; FFx1~~phix*FFx1; FFy1~~phiy*FFy1; FFx1~~phixy*FFy1;
	FFy2~ betay*FFy1+gammay*FFx1; FFx2~ betax*FFx1+gammax*FFy1
	FFy3~ betay*FFy2+gammay*FFx2; FFx3~ betax*FFx2+gammax*FFy2
	FFy4~ betay*FFy3+gammay*FFx3; FFx4~ betax*FFx3+gammax*FFy3
	FFy5~ betay*FFy4+gammay*FFx4; FFx5~ betax*FFx4+gammax*FFy4
	FFy6~ betay*FFy5+gammay*FFx5; FFx6~ betax*FFx5+gammax*FFy5
	FFy7~ betay*FFy6+gammay*FFx6; FFx7~ betax*FFx6+gammax*FFy6
	FFy8~ betay*FFy7+gammay*FFx7; FFx8~ betax*FFx7+gammax*FFy7
	FFx2~~Omegax*FFx2; FFx3~~Omegax*FFx3; FFx4~~Omegax*FFx4; FFx5~~Omegax*FFx5; FFx6~~Omegax*FFx6; FFx7~~Omegax*FFx7; FFx8~~Omegax*FFx8
	FFy2~~Omegay*FFy2; FFy3~~Omegay*FFy3; FFy4~~Omegay*FFy4; FFy5~~Omegay*FFy5; FFy6~~Omegay*FFy6; FFy7~~Omegay*FFy7; FFy8~~Omegay*FFy8
	FFx2~~Omegaxy*FFy2; FFx3~~Omegaxy*FFy3; FFx4~~Omegaxy*FFy4; FFx5~~Omegaxy*FFy5; FFx6~~Omegaxy*FFy6; FFx7~~Omegaxy*FFy7;
	FFx8~~Omegaxy*FFy8
	FFy1 =~ 1*y1; FFy2 =~ 1*y2; FFy3 =~ 1*y3; FFy4 =~ 1*y4; FFy5 =~ 1*y5; FFy6 =~ 1*y6; FFy7 =~ 1*y7; FFy8 =~ 1*y8
	FFx1 =~ 1*x1; FFx2 =~ 1*x2; FFx3 =~ 1*x3; FFx4 =~ 1*x4; FFx5 =~ 1*x5; FFx6 =~ 1*x6; FFx7 =~ 1*x7; FFx8 =~ 1*x8
'
```

#Lavaan code for the STARTS

#STARTS(T=4)

```
STARTST4 <- '
x1~mux1*1; y1~muy1*1; x2~mux2*1; y2~muy2*1; x3~mux3*1; y3~muy3*1; x4~mux4*1; y4~muy4*1;
Tx~~0*FFx1;Tx~~0*FFy1; Ty~~0*FFx1;Ty~~0*FFy1;
Tx=~1*x1+1*x2+1*x3+1*x4; Ty=~1*y1+1*y2+1*y3+1*y4
Tx~0*1; Ty~0*1; Tx~~taux*Tx; Ty~~tauy*Ty; Tx~~tauxy*Ty
x1 ~~ psix*x1; x2 ~~ psix*x2; x3 ~~ psix*x3; x4 ~~ psix*x4
y1 ~~ psiy*y1; y2 ~~ psiy*y2; y3 ~~ psiy*y3; y4 ~~ psiy*y4
y1 ~~ psixy*x1; y2 ~~ psixy*x2; y3 ~~ psixy*x3; y4 ~~ psixy*x4
FFx1~0*1;FFy1~0*1; FFx1~~phix*FFx1; FFy1~~phiy*FFy1; FFx1~~phixy*FFy1;
FFy2~ betay*FFy1+gammay*FFx1; FFx2~ betax*FFx1+gammax*FFy1
FFy3~ betay*FFy2+gammay*FFx2; FFx3~ betax*FFx2+gammax*FFy2
FFy4~ betay*FFy3+gammay*FFx3; FFx4~ betax*FFx3+gammax*FFy3
FFx2~~Omegax*FFx2; FFx3~~Omegax*FFx3; FFx4~~Omegax*FFx4;
FFy2~~Omegay*FFy2; FFy3~~Omegay*FFy3; FFy4~~Omegay*FFy4;
FFx2~~Omegaxy*FFy2; FFx3~~Omegaxy*FFy3; FFx4~~Omegaxy*FFy4;
FFy1 =~ 1*y1; FFy2 =~ 1*y2; FFy3 =~ 1*y3; FFy4 =~ 1*y4
FFx1 =~ 1*x1; FFx2 =~ 1*x2; FFx3 =~ 1*x3; FFx4 =~ 1*x4

'
```

#STARTS(T=6)

```
STARTST6 <- '
x1~mux1*1; y1~muy1*1; x2~mux2*1; y2~muy2*1; x3~mux3*1; y3~muy3*1; x4~mux4*1; y4~muy4*1;
x5~mux5*1; y5~muy5*1; x6~mux6*1; y6~muy6*1
Tx~~0*FFx1;Tx~~0*FFy1; Ty~~0*FFx1;Ty~~0*FFy1;
Tx=~1*x1+1*x2+1*x3+1*x4+1*x5+1*x6; Ty=~1*y1+1*y2+1*y3+1*y4+1*y5+1*y6
Tx~0*1; Ty~0*1; Tx~~taux*Tx; Ty~~tauy*Ty; Tx~~tauxy*Ty
x1 ~~ psix*x1; x2 ~~ psix*x2; x3 ~~ psix*x3; x4 ~~ psix*x4; x5 ~~ psix*x5; x6 ~~ psix*x6
y1 ~~ psiy*y1; y2 ~~ psiy*y2; y3 ~~ psiy*y3; y4 ~~ psiy*y4; y5 ~~ psiy*y5; y6 ~~ psiy*y6
y1 ~~ psixy*x1; y2 ~~ psixy*x2; y3 ~~ psixy*x3; y4 ~~ psixy*x4; y5 ~~ psixy*x5; y6 ~~ psixy*x6
FFx1~0*1;FFy1~0*1; FFx1~~phix*FFx1; FFy1~~phiy*FFy1; FFx1~~phixy*FFy1;
FFy2~ betay*FFy1+gammay*FFx1; FFx2~ betax*FFx1+gammax*FFy1
FFy3~ betay*FFy2+gammay*FFx2; FFx3~ betax*FFx2+gammax*FFy2
FFy4~ betay*FFy3+gammay*FFx3; FFx4~ betax*FFx3+gammax*FFy3
FFy5~ betay*FFy4+gammay*FFx4; FFx5~ betax*FFx4+gammax*FFy4
FFy6~ betay*FFy5+gammay*FFx5; FFx6~ betax*FFx5+gammax*FFy5
FFx2~~Omegax*FFx2; FFx3~~Omegax*FFx3; FFx4~~Omegax*FFx4; FFx5~~Omegax*FFx5; FFx6~~Omegax*FFx6
FFy2~~Omegay*FFy2; FFy3~~Omegay*FFy3; FFy4~~Omegay*FFy4; FFy5~~Omegay*FFy5; FFy6~~Omegay*FFy6
FFx2~~Omegaxy*FFy2; FFx3~~Omegaxy*FFy3; FFx4~~Omegaxy*FFy4; FFx5~~Omegaxy*FFy5; FFx6~~Omegaxy*FFy6
FFy1 =~ 1*y1; FFy2 =~ 1*y2; FFy3 =~ 1*y3; FFy4 =~ 1*y4; FFy5 =~ 1*y5; FFy6 =~ 1*y6
FFx1 =~ 1*x1; FFx2 =~ 1*x2; FFx3 =~ 1*x3; FFx4 =~ 1*x4; FFx5 =~ 1*x5; FFx6 =~ 1*x6
'
```

#STARTS(T=8)

```
STARTST8 <- '
x1~mux1*1; y1~muy1*1; x2~mux2*1; y2~muy2*1; x3~mux3*1; y3~muy3*1; x4~mux4*1; y4~muy4*1;
x5~mux5*1; y5~muy5*1; x6~mux6*1; y6~muy6*1; x7~mux7*1; y7~muy7*1; x8~mux8*1; y8~muy8*1;
Tx~~0*FFx1;Tx~~0*FFy1; Ty~~0*FFx1;Ty~~0*FFy1;
Tx=~1*x1+1*x2+1*x3+1*x4+1*x5+1*x6+1*x7+1*x8; Ty=~1*y1+1*y2+1*y3+1*y4+1*y5+1*y6+1*y7+1*y8
Tx~0*1; Ty~0*1; Tx~~taux*Tx; Ty~~tauy*Ty; Tx~~tauxy*Ty
x1 ~~ psix*x1; x2 ~~ psix*x2; x3 ~~ psix*x3; x4 ~~ psix*x4; x5 ~~ psix*x5; x6 ~~ psix*x6; x7 ~~ psix*x7; x8 ~~ psix*x8
y1 ~~ psiy*y1; y2 ~~ psiy*y2; y3 ~~ psiy*y3; y4 ~~ psiy*y4; y5 ~~ psiy*y5; y6 ~~ psiy*y6; y7 ~~ psiy*y7; y8 ~~ psiy*y8
y1 ~~ psixy*x1; y2 ~~ psixy*x2; y3 ~~ psixy*x3; y4 ~~ psixy*x4; y5 ~~ psixy*x5; y6 ~~ psixy*x6; y7 ~~ psixy*x7; y8 ~~ psixy*x8
FFx1~0*1;FFy1~0*1; FFx1~~phix*FFx1; FFy1~~phiy*FFy1; FFx1~~phixy*FFy1;
FFy2~ betay*FFy1+gammay*FFx1; FFx2~ betax*FFx1+gammax*FFy1
FFy3~ betay*FFy2+gammay*FFx2; FFx3~ betax*FFx2+gammax*FFy2
FFy4~ betay*FFy3+gammay*FFx3; FFx4~ betax*FFx3+gammax*FFy3
FFy5~ betay*FFy4+gammay*FFx4; FFx5~ betax*FFx4+gammax*FFy4
FFy6~ betay*FFy5+gammay*FFx5; FFx6~ betax*FFx5+gammax*FFy5
FFy7~ betay*FFy6+gammay*FFx6; FFx7~ betax*FFx6+gammax*FFy6
FFy8~ betay*FFy7+gammay*FFx7; FFx8~ betax*FFx7+gammax*FFy7
FFx2~~Omegax*FFx2; FFx3~~Omegax*FFx3; FFx4~~Omegax*FFx4; FFx5~~Omegax*FFx5; FFx6~~Omegax*FFx6; FFx7~~Omegax*FFx7; FFx8~~Omegax*FFx8
FFy2~~Omegay*FFy2; FFy3~~Omegay*FFy3; FFy4~~Omegay*FFy4; FFy5~~Omegay*FFy5; FFy6~~Omegay*FFy6; FFy7~~Omegay*FFy7; FFy8~~Omegay*FFy8
FFx2~~Omegaxy*FFy2; FFx3~~Omegaxy*FFy3; FFx4~~Omegaxy*FFy4; FFx5~~Omegaxy*FFy5; FFx6~~Omegaxy*FFy6; FFx7~~Omegaxy*FFy7;
FFx8~~Omegaxy*FFy8
FFy1 =~ 1*y1; FFy2 =~ 1*y2; FFy3 =~ 1*y3; FFy4 =~ 1*y4; FFy5 =~ 1*y5; FFy6 =~ 1*y6; FFy7 =~ 1*y7; FFy8 =~ 1*y8
FFx1 =~ 1*x1; FFx2 =~ 1*x2; FFx3 =~ 1*x3; FFx4 =~ 1*x4; FFx5 =~ 1*x5; FFx6 =~ 1*x6; FFx7 =~ 1*x7; FFx8 =~ 1*x8
'
```

2. Simulation codes for frequency of improper solutions

#Simulation Code when the CLPM is the data generation model (T=4)

```
SIMULATIONCLPMT4<-function(Repeat){
library(MASS) ;require("lavaan");RESULT<-matrix(rep(0,3^2*4*44),3^2*4,44)
options(warn=-1)
for(bbbb in 1:3){ #Specifying beta.(autoregressive parameter)
for(cccc in 1:3){ #Specifying psi (measurement error variances)
for(dddd in 1:4){ #specifying sample size

TT<-4;c1<-0;c2<-0    #Specifying the number of time points (T=4) and data generation model as the CLPM (c1 specifes measurent error and c2 specifies trait factor)

NN<-c(200,400,800,1600)[dddd] #sample size

#Specifying parameters
psix2<-c(0.2,0.5,0.8)[cccc];psiy2<-c(0.2,0.5,0.8)[cccc];psixy<-0.2*psix2 #measurement error (co)variances
phix12<-1-psix2; phiy12<-1-psiy2;phix1y1<-0.2*phix12 #phi1 is the variance at the first time point
phiTx2<-phix12*0.5; phiTy2<-phiy12*0.5;phiTxTy<-0.2*phiTx2 #specifying the trait factor (co)variances
phix1Tx<-0.1*sqrt(phix12*phiTx2);phix1Ty<-0.1*sqrt(phix12*phiTy2) #specifying correlations
phiy1Tx<-0.1*sqrt(phiy12*phiTx2);phiy1Ty<-0.1*sqrt(phiy12*phiTy2) #specifying correlations
betax<-c(0.5,0.7,0.9)[bbbb]; betay<-c(0.5,0.7,0.9)[bbbb]; gammax<-0.2;gammay<-0.2 #autoregressive and cross-lagged parameters
omegax<-0.2; omegay<-0.2; omegaxy<-0.2*omegax #residual (co)variances
mux1<-0; mux2<-1; mux3<-2; mux4<-3; mux5<-4; mux6<-5; mux7<-6; mux8<-7 #temporal group means
muy1<-0; muy2<-1; muy3<-2; muy4<-3; muy5<-4; muy6<-5; muy7<-6; muy8<-7
muTx<-0; muTy<-0 #trait factor means (=0)

for(ttt in 1:Repeat){

Factors<-mvrnorm(NN,c(mux1,muy1,muTx,muTy),
matrix(c(
phix12,phix1y1,phix1Tx,phix1Ty,
phix1y1,phiy12,phiy1Tx,phiy1Ty,
phix1Tx,phiy1Tx,phiTx2,phiTxTy,
phix1Ty,phiy1Ty,phiTxTy,phiTy2
),4,4))

#DATA generation
FFx1<-Factors[,1]; FFy1<-Factors[,2]; Tx<-Factors[,3]; Ty<-Factors[,4];
DT1<-cbind(FFx1,FFy1)+c1*mvrnorm(NN, rep(0,2),matrix(c(psix2,psixy,psixy,psiy2),2,2))+(c(mux1,muy1)+ c2*cbind(Tx,Ty))

FF2<- cbind(betax*FFx1+gammax*FFy1, betay*FFy1+gammay*FFx1)+mvrnorm(NN,rep(0,2), matrix(c(omegax,omegaxy,omegaxy,omegay),2,2));
FFx2<-FF2[,1]; FFy2<-FF2[,2]
DT2<-FF2+c1*mvrnorm(NN, rep(0,2),matrix(c(psix2,psixy,psixy,psiy2),2,2))+(c(mux2,muy2)+ c2*cbind(Tx,Ty))

FF3<- cbind(betax*FFx2+gammax*FFy2, betay*FFy2+gammay*FFx2)+mvrnorm(NN,rep(0,2), matrix(c(omegax,omegaxy,omegaxy,omegay),2,2));
FFx3<-FF3[,1]; FFy3<-FF3[,2]
DT3<-FF3+c1*mvrnorm(NN, rep(0,2),matrix(c(psix2,psixy,psixy,psiy2),2,2))+(c(mux3,muy3)+ c2*cbind(Tx,Ty))

FF4<- cbind(betax*FFx3+gammax*FFy3, betay*FFy3+gammay*FFx3)+mvrnorm(NN,rep(0,2), matrix(c(omegax,omegaxy,omegaxy,omegay),2,2));
FFx4<-FF4[,1]; FFy4<-FF4[,2]
DT4<-FF4+c1*mvrnorm(NN, rep(0,2),matrix(c(psix2,psixy,psixy,psiy2),2,2))+(c(mux4,muy4)+ c2*cbind(Tx,Ty))

FF5<- cbind(betax*FFx4+gammax*FFy4, betay*FFy4+gammay*FFx4)+mvrnorm(NN,rep(0,2), matrix(c(omegax,omegaxy,omegaxy,omegay),2,2));
FFx5<-FF5[,1]; FFy5<-FF5[,2]
DT5<-FF5+c1*mvrnorm(NN, rep(0,2),matrix(c(psix2,psixy,psixy,psiy2),2,2))+(c(mux5,muy5)+ c2*cbind(Tx,Ty))

FF6<- cbind(betax*FFx5+gammax*FFy5, betay*FFy5+gammay*FFx5)+mvrnorm(NN,rep(0,2), matrix(c(omegax,omegaxy,omegaxy,omegay),2,2));
FFx6<-FF6[,1]; FFy6<-FF6[,2]
DT6<-FF6+c1*mvrnorm(NN, rep(0,2),matrix(c(psix2,psixy,psixy,psiy2),2,2))+(c(mux6,muy6)+ c2*cbind(Tx,Ty))

FF7<- cbind(betax*FFx6+gammax*FFy6, betay*FFy6+gammay*FFx6)+mvrnorm(NN,rep(0,2), matrix(c(omegax,omegaxy,omegaxy,omegay),2,2));
FFx7<-FF7[,1]; FFy7<-FF7[,2]
DT7<-FF7+c1*mvrnorm(NN, rep(0,2),matrix(c(psix2,psixy,psixy,psiy2),2,2))+(c(mux7,muy7)+ c2*cbind(Tx,Ty))

FF8<- cbind(betax*FFx7+gammax*FFy7, betay*FFy7+gammay*FFx7)+mvrnorm(NN,rep(0,2), matrix(c(omegax,omegaxy,omegaxy,omegay),2,2));
FFx8<-FF8[,1]; FFy8<-FF8[,2]
```

```
DT8<-FF8+c1*mvrnorm(NN, rep(0,2),matrix(c(psix2,psixy,psixy,psiy2),2,2))+(c(mux8,muy8)+ c2*cbind(Tx,Ty))

Data<-data.frame(cbind(DT1,DT2,DT3,DT4,DT5,DT6,DT7,DT8))
colnames(Data)<-c("x1","y1","x2","y2","x3","y3","x4","y4","x5","y5","x6","y6","x7","y7","x8","y8")
DATA<-Data[,1:(2*TT)]

RESULT[4*3*(cccc-1)+4*(bbbb-1)+dddd,1]<- 0
RESULT[4*3*(cccc-1)+4*(bbbb-1)+dddd,2]<- betax
RESULT[4*3*(cccc-1)+4*(bbbb-1)+dddd,3]<- psix2
RESULT[4*3*(cccc-1)+4*(bbbb-1)+dddd,4]<- NN

#DATA analysis and count the frequency of the improper solutions
fit<- suppressMessages(suppressWarnings (lavaan(CLPMT4, data = DATA))) #change the red-highlighted part for different T
COU<-sign(sum(diag(inspect(fit, "coefficients")$psi)<0)+sum(diag(inspect(fit, "coefficients")$theta)<0)+sum(det(fitted(fit)$cov)<0))
COU2<-(inspect(fit, "coefficients")$beta[2,1]-betay); COU3<-(inspect(fit, "coefficients")$beta[2+TT,1]-gammax)
COU4<-(inspect(fit, "coefficients")$beta[2,1]-betay)^2; COU5<-(inspect(fit, "coefficients")$beta[2+TT,1]-gammax)^2
RESULT[4*3*(cccc-1)+4*(bbbb-1)+dddd,5]<- RESULT[4*3*(cccc-1)+4*(bbbb-1)+dddd,5]+COU/Repeat
RESULT[4*3*(cccc-1)+4*(bbbb-1)+dddd,6]<- RESULT[4*3*(cccc-1)+4*(bbbb-1)+dddd,6]+COU2/Repeat
RESULT[4*3*(cccc-1)+4*(bbbb-1)+dddd,7]<- RESULT[4*3*(cccc-1)+4*(bbbb-1)+dddd,7]+COU3/Repeat
RESULT[4*3*(cccc-1)+4*(bbbb-1)+dddd,8]<- RESULT[4*3*(cccc-1)+4*(bbbb-1)+dddd,8]+COU4/Repeat
RESULT[4*3*(cccc-1)+4*(bbbb-1)+dddd,9]<- RESULT[4*3*(cccc-1)+4*(bbbb-1)+dddd,9]+COU5/Repeat

fit<- suppressMessages(suppressWarnings (lavaan(RICLPMT4, data = DATA)) ) #change the red-highlighted part for different T
COU<-sign(sum(diag(inspect(fit, "coefficients")$psi)<0)+sum(diag(inspect(fit, "coefficients")$theta)<0)+sum(det(fitted(fit)$cov)<0))
COU2<-(inspect(fit, "coefficients")$beta[4,3]-betay); COU3<-(inspect(fit, "coefficients")$beta[4+TT,3]-gammax)
COU4<-(inspect(fit, "coefficients")$beta[4,3]-betay)^2; COU5<-(inspect(fit, "coefficients")$beta[4+TT,3]-gammax)^2
RESULT[4*3*(cccc-1)+4*(bbbb-1)+dddd,10]<- RESULT[4*3*(cccc-1)+4*(bbbb-1)+dddd,10]+COU/Repeat
RESULT[4*3*(cccc-1)+4*(bbbb-1)+dddd,11]<- RESULT[4*3*(cccc-1)+4*(bbbb-1)+dddd,11]+COU2/Repeat
RESULT[4*3*(cccc-1)+4*(bbbb-1)+dddd,12]<- RESULT[4*3*(cccc-1)+4*(bbbb-1)+dddd,12]+COU3/Repeat
RESULT[4*3*(cccc-1)+4*(bbbb-1)+dddd,13]<- RESULT[4*3*(cccc-1)+4*(bbbb-1)+dddd,13]+COU4/Repeat
RESULT[4*3*(cccc-1)+4*(bbbb-1)+dddd,14]<- RESULT[4*3*(cccc-1)+4*(bbbb-1)+dddd,14]+COU5/Repeat

fit<- suppressMessages(suppressWarnings(lavaan(STARTST4, data = DATA)) ) #change the red-highlighted part for different T
COU<-sign(sum(diag(inspect(fit, "coefficients")$psi)<0)+sum(diag(inspect(fit, "coefficients")$theta)<0)+sum(det(fitted(fit)$cov)<0))
COU2<-(inspect(fit, "coefficients")$beta[4,3]-betay); COU3<-(inspect(fit, "coefficients")$beta[4+TT,3]-gammax)
COU4<-(inspect(fit, "coefficients")$beta[4,3]-betay)^2; COU5<-(inspect(fit, "coefficients")$beta[4+TT,3]-gammax)^2
RESULT[4*3*(cccc-1)+4*(bbbb-1)+dddd,15]<- RESULT[4*3*(cccc-1)+4*(bbbb-1)+dddd,15]+COU/Repeat
RESULT[4*3*(cccc-1)+4*(bbbb-1)+dddd,16]<- RESULT[4*3*(cccc-1)+4*(bbbb-1)+dddd,16]+COU2/Repeat
RESULT[4*3*(cccc-1)+4*(bbbb-1)+dddd,17]<- RESULT[4*3*(cccc-1)+4*(bbbb-1)+dddd,17]+COU3/Repeat
RESULT[4*3*(cccc-1)+4*(bbbb-1)+dddd,18]<- RESULT[4*3*(cccc-1)+4*(bbbb-1)+dddd,18]+COU4/Repeat
RESULT[4*3*(cccc-1)+4*(bbbb-1)+dddd,19]<- RESULT[4*3*(cccc-1)+4*(bbbb-1)+dddd,19]+COU5/Repeat

};};};}

write.csv(RESULT,"Specifying an appropriate directory and file name here")
}
```

#Simulation Code when the RI-CLPM is the data generation model (T=4)

```
SIMULATIONRICLPMT4<-function(Repeat){
library(MASS) ;require("lavaan");RESULT<-matrix(rep(0,3^2*4*44),3^2*4,44)
options(warn=-1)
for(bbbb in 1:3){ #Specifying beta.(autoregressive parameter)
for(cccc in 1:3){ #Specifying psi (measurement error variances)
for(dddd in 1:4){ #specifying sample size

TT<-4;c1<-0;c2<-1    #Specifying the number of time points (T=4) and data generation model as the RI-CLPM (c1 specifes measurent error and c2 specifies trait factor)

NN<-c(200,400,800,1600)[dddd] #sample size

#Specifying parameters
psix2<-c(0.2,0.5,0.8)[cccc];psiy2<-c(0.2,0.5,0.8)[cccc];psixy<-0.2*psix2 #measurement error (co)variances
phix12<-1-psix2; phiy12<-1-psiy2;phix1y1<-0.2*phix12 #phi1 is the variance at the first time point
phiTx2<-phix12*0.5; phiTy2<-phiy12*0.5;phiTxTy<-0.2*phiTx2 #specifying the trait factor (co)variances
phix1Tx<-0.1*sqrt(phix12*phiTx2);phix1Ty<-0.1*sqrt(phix12*phiTy2) #specifying correlations
phiy1Tx<-0.1*sqrt(phiy12*phiTx2);phiy1Ty<-0.1*sqrt(phiy12*phiTy2) #specifying correlations
betax<-c(0.5,0.7,0.9)[bbbb]; betay<-c(0.5,0.7,0.9)[bbbb]; gammax<-0.2;gammay<-0.2 #autoregressive and cross-lagged parameters
omegax<-0.2; omegay<-0.2; omegaxy<-0.2*omegax #residual (co)variances
mux1<-0; mux2<-1; mux3<-2; mux4<-3; mux5<-4; mux6<-5; mux7<-6; mux8<-7 #temporal group means
muy1<-0; muy2<-1; muy3<-2; muy4<-3; muy5<-4; muy6<-5; muy7<-6; muy8<-7
muTx<-0; muTy<-0 #trait factor means (=0)

for(ttt in 1:Repeat){

Factors<-mvrnorm(NN,c(mux1,muy1,muTx,muTy),
matrix(c(
phix12,phix1y1,phix1Tx,phix1Ty,
phix1y1,phiy12,phiy1Tx,phiy1Ty,
phix1Tx,phiy1Tx,phiTx2,phiTxTy,
phix1Ty,phiy1Ty,phiTxTy,phiTy2
),4,4))

#DATA generation
FFx1<-Factors[,1]; FFy1<-Factors[,2]; Tx<-Factors[,3]; Ty<-Factors[,4];

DT1<-cbind(FFx1,FFy1)+c1*mvrnorm(NN, rep(0,2),matrix(c(psix2,psixy,psixy,psiy2),2,2))+(c(mux1,muy1)+ c2*cbind(Tx,Ty))

FF2<- cbind(betax*FFx1+gammax*FFy1, betay*FFy1+gammay*FFx1)+mvrnorm(NN,rep(0,2), matrix(c(omegax,omegaxy,omegaxy,omegay),2,2));
FFx2<-FF2[,1]; FFy2<-FF2[,2]
DT2<-FF2+c1*mvrnorm(NN, rep(0,2),matrix(c(psix2,psixy,psixy,psiy2),2,2))+(c(mux2,muy2)+ c2*cbind(Tx,Ty))

FF3<- cbind(betax*FFx2+gammax*FFy2, betay*FFy2+gammay*FFx2)+mvrnorm(NN,rep(0,2), matrix(c(omegax,omegaxy,omegaxy,omegay),2,2));
FFx3<-FF3[,1]; FFy3<-FF3[,2]
DT3<-FF3+c1*mvrnorm(NN, rep(0,2),matrix(c(psix2,psixy,psixy,psiy2),2,2))+(c(mux3,muy3)+ c2*cbind(Tx,Ty))

FF4<- cbind(betax*FFx3+gammax*FFy3, betay*FFy3+gammay*FFx3)+mvrnorm(NN,rep(0,2), matrix(c(omegax,omegaxy,omegaxy,omegay),2,2));
FFx4<-FF4[,1]; FFy4<-FF4[,2]
DT4<-FF4+c1*mvrnorm(NN, rep(0,2),matrix(c(psix2,psixy,psixy,psiy2),2,2))+(c(mux4,muy4)+ c2*cbind(Tx,Ty))

FF5<- cbind(betax*FFx4+gammax*FFy4, betay*FFy4+gammay*FFx4)+mvrnorm(NN,rep(0,2), matrix(c(omegax,omegaxy,omegaxy,omegay),2,2));
FFx5<-FF5[,1]; FFy5<-FF5[,2]
DT5<-FF5+c1*mvrnorm(NN, rep(0,2),matrix(c(psix2,psixy,psixy,psiy2),2,2))+(c(mux5,muy5)+ c2*cbind(Tx,Ty))

FF6<- cbind(betax*FFx5+gammax*FFy5, betay*FFy5+gammay*FFx5)+mvrnorm(NN,rep(0,2), matrix(c(omegax,omegaxy,omegaxy,omegay),2,2));
FFx6<-FF6[,1]; FFy6<-FF6[,2]
DT6<-FF6+c1*mvrnorm(NN, rep(0,2),matrix(c(psix2,psixy,psixy,psiy2),2,2))+(c(mux6,muy6)+ c2*cbind(Tx,Ty))

FF7<- cbind(betax*FFx6+gammax*FFy6, betay*FFy6+gammay*FFx6)+mvrnorm(NN,rep(0,2), matrix(c(omegax,omegaxy,omegaxy,omegay),2,2));
FFx7<-FF7[,1]; FFy7<-FF7[,2]
DT7<-FF7+c1*mvrnorm(NN, rep(0,2),matrix(c(psix2,psixy,psixy,psiy2),2,2))+(c(mux7,muy7)+ c2*cbind(Tx,Ty))

FF8<- cbind(betax*FFx7+gammax*FFy7, betay*FFy7+gammay*FFx7)+mvrnorm(NN,rep(0,2), matrix(c(omegax,omegaxy,omegaxy,omegay),2,2));
FFx8<-FF8[,1]; FFy8<-FF8[,2]
DT8<-FF8+c1*mvrnorm(NN, rep(0,2),matrix(c(psix2,psixy,psixy,psiy2),2,2))+(c(mux8,muy8)+ c2*cbind(Tx,Ty))
```

```
Data<-data.frame(cbind(DT1,DT2,DT3,DT4,DT5,DT6,DT7,DT8))
colnames(Data)<-c("x1","y1","x2","y2","x3","y3","x4","y4","x5","y5","x6","y6","x7","y7","x8","y8")
DATA<-Data[,1:(2*TT)]

RESULT[4*3*(cccc-1)+4*(bbbb-1)+dddd,1]<- 0
RESULT[4*3*(cccc-1)+4*(bbbb-1)+dddd,2]<- betax
RESULT[4*3*(cccc-1)+4*(bbbb-1)+dddd,3]<- psix2
RESULT[4*3*(cccc-1)+4*(bbbb-1)+dddd,4]<- NN

#DATA analysis and count the frequency of the improper solutions
fit<- suppressMessages(suppressWarnings (lavaan(CLPMT4, data = DATA))) #change the red-highlighted part for different T
COU<-sign(sum(diag(inspect(fit, "coefficients")$psi)<0)+sum(diag(inspect(fit, "coefficients")$theta)<0)+sum(det(fitted(fit)$cov)<0))
COU2<-(inspect(fit, "coefficients")$beta[2,1]-betay); COU3<-(inspect(fit, "coefficients")$beta[2+TT,1]-gammax)
COU4<-(inspect(fit, "coefficients")$beta[2,1]-betay)^2; COU5<-(inspect(fit, "coefficients")$beta[2+TT,1]-gammax)^2
RESULT[4*3*(cccc-1)+4*(bbbb-1)+dddd,5]<- RESULT[4*3*(cccc-1)+4*(bbbb-1)+dddd,5]+COU/Repeat
RESULT[4*3*(cccc-1)+4*(bbbb-1)+dddd,6]<- RESULT[4*3*(cccc-1)+4*(bbbb-1)+dddd,6]+COU2/Repeat
RESULT[4*3*(cccc-1)+4*(bbbb-1)+dddd,7]<- RESULT[4*3*(cccc-1)+4*(bbbb-1)+dddd,7]+COU3/Repeat
RESULT[4*3*(cccc-1)+4*(bbbb-1)+dddd,8]<- RESULT[4*3*(cccc-1)+4*(bbbb-1)+dddd,8]+COU4/Repeat
RESULT[4*3*(cccc-1)+4*(bbbb-1)+dddd,9]<- RESULT[4*3*(cccc-1)+4*(bbbb-1)+dddd,9]+COU5/Repeat

fit<- suppressMessages(suppressWarnings (lavaan(RICLPMT4, data = DATA)) ) #change the red-highlighted part for different T
COU<-sign(sum(diag(inspect(fit, "coefficients")$psi)<0)+sum(diag(inspect(fit, "coefficients")$theta)<0)+sum(det(fitted(fit)$cov)<0))
COU2<-(inspect(fit, "coefficients")$beta[4,3]-betay); COU3<-(inspect(fit, "coefficients")$beta[4+TT,3]-gammax)
COU4<-(inspect(fit, "coefficients")$beta[4,3]-betay)^2; COU5<-(inspect(fit, "coefficients")$beta[4+TT,3]-gammax)^2
RESULT[4*3*(cccc-1)+4*(bbbb-1)+dddd,10]<- RESULT[4*3*(cccc-1)+4*(bbbb-1)+dddd,10]+COU/Repeat
RESULT[4*3*(cccc-1)+4*(bbbb-1)+dddd,11]<- RESULT[4*3*(cccc-1)+4*(bbbb-1)+dddd,11]+COU2/Repeat
RESULT[4*3*(cccc-1)+4*(bbbb-1)+dddd,12]<- RESULT[4*3*(cccc-1)+4*(bbbb-1)+dddd,12]+COU3/Repeat
RESULT[4*3*(cccc-1)+4*(bbbb-1)+dddd,13]<- RESULT[4*3*(cccc-1)+4*(bbbb-1)+dddd,13]+COU4/Repeat
RESULT[4*3*(cccc-1)+4*(bbbb-1)+dddd,14]<- RESULT[4*3*(cccc-1)+4*(bbbb-1)+dddd,14]+COU5/Repeat

fit<- suppressMessages(suppressWarnings(lavaan(STARTST4, data = DATA)) ) #change the red-highlighted part for different T
COU<-sign(sum(diag(inspect(fit, "coefficients")$psi)<0)+sum(diag(inspect(fit, "coefficients")$theta)<0)+sum(det(fitted(fit)$cov)<0))
COU2<-(inspect(fit, "coefficients")$beta[4,3]-betay); COU3<-(inspect(fit, "coefficients")$beta[4+TT,3]-gammax)
COU4<-(inspect(fit, "coefficients")$beta[4,3]-betay)^2; COU5<-(inspect(fit, "coefficients")$beta[4+TT,3]-gammax)^2
RESULT[4*3*(cccc-1)+4*(bbbb-1)+dddd,15]<- RESULT[4*3*(cccc-1)+4*(bbbb-1)+dddd,15]+COU/Repeat
RESULT[4*3*(cccc-1)+4*(bbbb-1)+dddd,16]<- RESULT[4*3*(cccc-1)+4*(bbbb-1)+dddd,16]+COU2/Repeat
RESULT[4*3*(cccc-1)+4*(bbbb-1)+dddd,17]<- RESULT[4*3*(cccc-1)+4*(bbbb-1)+dddd,17]+COU3/Repeat
RESULT[4*3*(cccc-1)+4*(bbbb-1)+dddd,18]<- RESULT[4*3*(cccc-1)+4*(bbbb-1)+dddd,18]+COU4/Repeat
RESULT[4*3*(cccc-1)+4*(bbbb-1)+dddd,19]<- RESULT[4*3*(cccc-1)+4*(bbbb-1)+dddd,19]+COU5/Repeat

};};};}

write.csv(RESULT,"Specifying an appropriate directory and file name here")
}
```

#Simulation Code when the STARTS model is the data generation model (T=4)

```
SIMULATIONSTARTST4<-function(Repeat){
library(MASS) ;require("lavaan");RESULT<-matrix(rep(0,3^2*4*44),3^2*4,44)
options(warn=-1)
for(bbbb in 1:3){ #Specifying beta.(autoregressive parameter)
for(cccc in 1:3){ #Specifying psi (measurement error variances)
for(dddd in 1:4){ #specifying sample size

TT<-4;c1<-1;c2<-1 #Specifying the number of time points (T=4) and data generation model as the STARTS model (c1-c4,alpha)

NN<-c(200,400,800,1600)[dddd] #sample size
```

#Specifying parameters

```
psix2<-c(0.2,0.5,0.8)[cccc];psiy2<-c(0.2,0.5,0.8)[cccc];psixy<-0.2*psix2 #measurement error (co)variances
phix12<-1-psix2; phiy12<-1-psiy2;phix1y1<-0.2*phix12 #phi1 is the variance at the first time point
phiTx2<-phix12*0.5; phiTy2<-phiy12*0.5;phiTxTy<-0.2*phiTx2 #specifying the trait factor (co)variances
phix1Tx<-0.1*sqrt(phix12*phiTx2);phix1Ty<-0.1*sqrt(phix12*phiTy2) #specifying correlations
phiy1Tx<-0.1*sqrt(phiy12*phiTx2);phiy1Ty<-0.1*sqrt(phiy12*phiTy2) #specifying correlations
betax<-c(0.5,0.7,0.9)[bbbb]; betay<-c(0.5,0.7,0.9)[bbbb]; gammax<-0.2;gammay<-0.2 #autoregressive and cross-lagged parameters
omegax<-0.2; omegay<-0.2; omegaxy<-0.2*omegax #residual (co)variances
mux1<-0; mux2<-1; mux3<-2; mux4<-3; mux5<-4; mux6<-5; mux7<-6; mux8<-7 #temporal group means
muy1<-0; muy2<-1; muy3<-2; muy4<-3; muy5<-4; muy6<-5; muy7<-6; muy8<-7
muTx<-0; muTy<-0 #trait factor means (=0)

for(ttt in 1:Repeat){

Factors<-mvrnorm(NN,c(mux1,muy1,muTx,muTy),
matrix(c(
phix12,phix1y1,phix1Tx,phix1Ty,
phix1y1,phiy12,phiy1Tx,phiy1Ty,
phix1Tx,phiy1Tx,phiTx2,phiTxTy,
phix1Ty,phiy1Ty,phiTxTy,phiTy2
),4,4))
```

#DATA generation

```
FFx1<-Factors[,1]; FFy1<-Factors[,2]; Tx<-Factors[,3]; Ty<-Factors[,4];

DT1<-cbind(FFx1,FFy1)+c1*mvrnorm(NN, rep(0,2),matrix(c(psix2,psixy,psixy,psiy2),2,2))+(c(mux1,muy1)+ c2*cbind(Tx,Ty))

FF2<- cbind(betax*FFx1+gammax*FFy1, betay*FFy1+gammay*FFx1)+mvrnorm(NN,rep(0,2), matrix(c(omegax,omegaxy,omegaxy,omegay),2,2));
FFx2<-FF2[,1]; FFy2<-FF2[,2]
DT2<-FF2+c1*mvrnorm(NN, rep(0,2),matrix(c(psix2,psixy,psixy,psiy2),2,2))+(c(mux2,muy2)+ c2*cbind(Tx,Ty))

FF3<- cbind(betax*FFx2+gammax*FFy2, betay*FFy2+gammay*FFx2)+mvrnorm(NN,rep(0,2), matrix(c(omegax,omegaxy,omegaxy,omegay),2,2));
FFx3<-FF3[,1]; FFy3<-FF3[,2]
DT3<-FF3+c1*mvrnorm(NN, rep(0,2),matrix(c(psix2,psixy,psixy,psiy2),2,2))+(c(mux3,muy3)+ c2*cbind(Tx,Ty))

FF4<- cbind(betax*FFx3+gammax*FFy3, betay*FFy3+gammay*FFx3)+mvrnorm(NN,rep(0,2), matrix(c(omegax,omegaxy,omegaxy,omegay),2,2));
FFx4<-FF4[,1]; FFy4<-FF4[,2]
DT4<-FF4+c1*mvrnorm(NN, rep(0,2),matrix(c(psix2,psixy,psixy,psiy2),2,2))+(c(mux4,muy4)+ c2*cbind(Tx,Ty))

FF5<- cbind(betax*FFx4+gammax*FFy4, betay*FFy4+gammay*FFx4)+mvrnorm(NN,rep(0,2), matrix(c(omegax,omegaxy,omegaxy,omegay),2,2));
FFx5<-FF5[,1]; FFy5<-FF5[,2]
DT5<-FF5+c1*mvrnorm(NN, rep(0,2),matrix(c(psix2,psixy,psixy,psiy2),2,2))+(c(mux5,muy5)+ c2*cbind(Tx,Ty))

FF6<- cbind(betax*FFx5+gammax*FFy5, betay*FFy5+gammay*FFx5)+mvrnorm(NN,rep(0,2), matrix(c(omegax,omegaxy,omegaxy,omegay),2,2));
FFx6<-FF6[,1]; FFy6<-FF6[,2]
DT6<-FF6+c1*mvrnorm(NN, rep(0,2),matrix(c(psix2,psixy,psixy,psiy2),2,2))+(c(mux6,muy6)+ c2*cbind(Tx,Ty))

FF7<- cbind(betax*FFx6+gammax*FFy6, betay*FFy6+gammay*FFx6)+mvrnorm(NN,rep(0,2), matrix(c(omegax,omegaxy,omegaxy,omegay),2,2));
FFx7<-FF7[,1]; FFy7<-FF7[,2]
DT7<-FF7+c1*mvrnorm(NN, rep(0,2),matrix(c(psix2,psixy,psixy,psiy2),2,2))+(c(mux7,muy7)+ c2*cbind(Tx,Ty))

FF8<- cbind(betax*FFx7+gammax*FFy7, betay*FFy7+gammay*FFx7)+mvrnorm(NN,rep(0,2), matrix(c(omegax,omegaxy,omegaxy,omegay),2,2));
FFx8<-FF8[,1]; FFy8<-FF8[,2]
DT8<-FF8+c1*mvrnorm(NN, rep(0,2),matrix(c(psix2,psixy,psixy,psiy2),2,2))+(c(mux8,muy8)+ c2*cbind(Tx,Ty))
```

```
Data<-data.frame(cbind(DT1,DT2,DT3,DT4,DT5,DT6,DT7,DT8))
colnames(Data)<-c("x1","y1","x2","y2","x3","y3","x4","y4","x5","y5","x6","y6","x7","y7","x8","y8")
DATA<-Data[,1:(2*TT)]

RESULT[4*3*(cccc-1)+4*(bbbb-1)+dddd,1]<- 0
RESULT[4*3*(cccc-1)+4*(bbbb-1)+dddd,2]<- betax
RESULT[4*3*(cccc-1)+4*(bbbb-1)+dddd,3]<- psix2
RESULT[4*3*(cccc-1)+4*(bbbb-1)+dddd,4]<- NN
```

#DATA analysis and count the frequency of the improper solutions

```
fit<- suppressMessages(suppressWarnings (lavaan(CLPMT4, data = DATA))) #change the red-highlighted part for different T
COU<-sign(sum(diag(inspect(fit, "coefficients")$psi)<0)+sum(diag(inspect(fit, "coefficients")$theta)<0)+sum(det(fitted(fit)$cov)<0))
COU2<-(inspect(fit, "coefficients")$beta[2,1]-betay); COU3<-(inspect(fit, "coefficients")$beta[2+TT,1]-gammax)
COU4<-(inspect(fit, "coefficients")$beta[2,1]-betay)^2; COU5<-(inspect(fit, "coefficients")$beta[2+TT,1]-gammax)^2
RESULT[4*3*(cccc-1)+4*(bbbb-1)+dddd,5]<- RESULT[4*3*(cccc-1)+4*(bbbb-1)+dddd,5]+COU/Repeat
RESULT[4*3*(cccc-1)+4*(bbbb-1)+dddd,6]<- RESULT[4*3*(cccc-1)+4*(bbbb-1)+dddd,6]+COU2/Repeat
RESULT[4*3*(cccc-1)+4*(bbbb-1)+dddd,7]<- RESULT[4*3*(cccc-1)+4*(bbbb-1)+dddd,7]+COU3/Repeat
RESULT[4*3*(cccc-1)+4*(bbbb-1)+dddd,8]<- RESULT[4*3*(cccc-1)+4*(bbbb-1)+dddd,8]+COU4/Repeat
RESULT[4*3*(cccc-1)+4*(bbbb-1)+dddd,9]<- RESULT[4*3*(cccc-1)+4*(bbbb-1)+dddd,9]+COU5/Repeat

fit<- suppressMessages(suppressWarnings (lavaan(RICLPMT4, data = DATA)) ) #change the red-highlighted part for different T
COU<-sign(sum(diag(inspect(fit, "coefficients")$psi)<0)+sum(diag(inspect(fit, "coefficients")$theta)<0)+sum(det(fitted(fit)$cov)<0))
COU2<-(inspect(fit, "coefficients")$beta[4,3]-betay); COU3<-(inspect(fit, "coefficients")$beta[4+TT,3]-gammax)
COU4<-(inspect(fit, "coefficients")$beta[4,3]-betay)^2; COU5<-(inspect(fit, "coefficients")$beta[4+TT,3]-gammax)^2
RESULT[4*3*(cccc-1)+4*(bbbb-1)+dddd,10]<- RESULT[4*3*(cccc-1)+4*(bbbb-1)+dddd,10]+COU/Repeat
RESULT[4*3*(cccc-1)+4*(bbbb-1)+dddd,11]<- RESULT[4*3*(cccc-1)+4*(bbbb-1)+dddd,11]+COU2/Repeat
RESULT[4*3*(cccc-1)+4*(bbbb-1)+dddd,12]<- RESULT[4*3*(cccc-1)+4*(bbbb-1)+dddd,12]+COU3/Repeat
RESULT[4*3*(cccc-1)+4*(bbbb-1)+dddd,13]<- RESULT[4*3*(cccc-1)+4*(bbbb-1)+dddd,13]+COU4/Repeat
RESULT[4*3*(cccc-1)+4*(bbbb-1)+dddd,14]<- RESULT[4*3*(cccc-1)+4*(bbbb-1)+dddd,14]+COU5/Repeat

fit<- suppressMessages(suppressWarnings(lavaan(STARTST4, data = DATA)) ) #change the red-highlighted part for different T
COU<-sign(sum(diag(inspect(fit, "coefficients")$psi)<0)+sum(diag(inspect(fit, "coefficients")$theta)<0)+sum(det(fitted(fit)$cov)<0))
COU2<-(inspect(fit, "coefficients")$beta[4,3]-betay); COU3<-(inspect(fit, "coefficients")$beta[4+TT,3]-gammax)
COU4<-(inspect(fit, "coefficients")$beta[4,3]-betay)^2; COU5<-(inspect(fit, "coefficients")$beta[4+TT,3]-gammax)^2
RESULT[4*3*(cccc-1)+4*(bbbb-1)+dddd,15]<- RESULT[4*3*(cccc-1)+4*(bbbb-1)+dddd,15]+COU/Repeat
RESULT[4*3*(cccc-1)+4*(bbbb-1)+dddd,16]<- RESULT[4*3*(cccc-1)+4*(bbbb-1)+dddd,16]+COU2/Repeat
RESULT[4*3*(cccc-1)+4*(bbbb-1)+dddd,17]<- RESULT[4*3*(cccc-1)+4*(bbbb-1)+dddd,17]+COU3/Repeat
RESULT[4*3*(cccc-1)+4*(bbbb-1)+dddd,18]<- RESULT[4*3*(cccc-1)+4*(bbbb-1)+dddd,18]+COU4/Repeat
RESULT[4*3*(cccc-1)+4*(bbbb-1)+dddd,19]<- RESULT[4*3*(cccc-1)+4*(bbbb-1)+dddd,19]+COU5/Repeat
};};};}

write.csv(RESULT,"Specifying an appropriate directory and file name here")
}
```

3. Simulation codes for statistical properties of estimates

#Simulation Code when the CLPM is the data generation model (T=4)

```
SIMULATIONCLPMT4<-function(Repeat){
library(MASS) ;require("lavaan");RESULT<-matrix(rep(0,3**3*2*43),3**3*2,43)
options(warn=-1)
for(aaaa in 1:3){ #Specifying cross-lagged parameters
for(bbbb in 1:2){ #Specifying auto regressive parameters
for(cccc in 1:3){ #Specifying measurement error variances
for(dddd in 1:3){ #Specifying sample size

TT<-4;c1<-0;c2<-0
NN<-c(200,600,1000)[dddd]
```

#Specifying parameters

```
psix2<-c(0.2,0.4,0.6)[cccc];psiy2<-c(0.2,0.4,0.6)[cccc];psixy<-0.2*psix2 #measurement error (co)variances
phix12<-1-psix2; phiy12<-1-psiy2;phix1y1<-0.2*phix12 #phi1 is the variance at the first time point
phiTx2<-phix12*0.5; phiTy2<-phiy12*0.5;phiTxTy<-0.2*phiTx2    #specifying the trait factor (co)variances
phix1Tx<-0.1*sqrt(phix12*phiTx2);phix1Ty<-0.1*sqrt(phix12*phiTy2) #specifying correlations
phiy1Tx<-0.1*sqrt(phiy12*phiTx2);phiy1Ty<-0.1*sqrt(phiy12*phiTy2) #specifying correlations
betax<-c(0.5,0.7)[bbbb];    betay<-c(0.5,0.7)[bbbb]; gammax<-c(0.0,0.1,0.2)[aaaa];gammay<- c(0.0,0.1,0.2)[aaaa] #autoregressive and cross-lagged parameters
omegax<-0.2; omegay<-0.2; omegaxy<-0.2*omegax    #residual (co)variances
mux1<-0; mux2<-1; mux3<-2; mux4<-3; mux5<-4; mux6<-5; mux7<-6; mux8<-7 #temporal group means
muy1<-0; muy2<-1; muy3<-2; muy4<-3; muy5<-4; muy6<-5; muy7<-6; muy8<-7
muTx<-0; muTy<-0 #trait factor means (=0)

ttt<-0
CLGSDES<-rep(0,Repeat);CLGES<- rep(0,Repeat);RICLGSDES<- rep(0,Repeat);RICLGES<- rep(0,Repeat);STGSDES<- rep(0,Repeat);STGES<- rep(0,Repeat)
CLGESbias<- rep(0,Repeat); RICLGESbias<- rep(0,Repeat);STGESbias<- rep(0,Repeat)
CLGESse<- rep(0,Repeat); RICLGESse<- rep(0,Repeat);STGESse<- rep(0,Repeat)
AIC1ES<- 0; AIC2ES<-0;AIC3ES<- 0; BIC1ES<- 0; BIC2ES<- 0;BIC3ES<- 0

sumSIGDiffCLRICLG1<- 0;sumSIGDiffCLRICLG2<- 0;sumSIGDiffCLRICLG3<- 0;sumSIGDiffCLRICLG4<-0
sumSIGDiffCLSTG1<- 0;sumSIGDiffCLSTG2<-0;sumSIGDiffCLSTG3<- 0;sumSIGDiffCLSTG4<-0
sumSIGDiffRICLSTG1<- 0;sumSIGDiffRICLSTG2<- 0;sumSIGDiffRICLSTG3<- 0;sumSIGDiffRICLSTG4<- 0
while(ttt<Repeat){
Factors<-mvrnorm(NN,c(mux1,muy1,muTx,muTy),
matrix(c(
phix12,phix1y1,phix1Tx,phix1Ty,
phix1y1,phiy12,phiy1Tx,phiy1Ty,
phix1Tx,phiy1Tx,phiTx2,phiTxTy,
phix1Ty,phiy1Ty,phiTxTy,phiTy2
),4,4))
```

#DATA generation

```
FFx1<-Factors[,1]; FFy1<-Factors[,2]; Tx<-Factors[,3]; Ty<-Factors[,4];
DT1<-cbind(FFx1,FFy1)+c1*mvrnorm(NN, rep(0,2),matrix(c(psix2,psixy,psixy,psiy2),2,2))+(c(mux1,muy1)+ c2*cbind(Tx,Ty))
FF2<- cbind(betax*FFx1+gammax*FFy1, betay*FFy1+gammay*FFx1)+mvrnorm(NN,rep(0,2), matrix(c(omegax,omegaxy,omegaxy,omegay),2,2));
FFx2<-FF2[,1]; FFy2<-FF2[,2]
DT2<-FF2+c1*mvrnorm(NN, rep(0,2),matrix(c(psix2,psixy,psixy,psiy2),2,2))+(c(mux2,muy2)+ c2*cbind(Tx,Ty))
FF3<- cbind(betax*FFx2+gammax*FFy2, betay*FFy2+gammay*FFx2)+mvrnorm(NN,rep(0,2), matrix(c(omegax,omegaxy,omegaxy,omegay),2,2));
FFx3<-FF3[,1]; FFy3<-FF3[,2]
DT3<-FF3+c1*mvrnorm(NN, rep(0,2),matrix(c(psix2,psixy,psixy,psiy2),2,2))+(c(mux3,muy3)+ c2*cbind(Tx,Ty))
FF4<- cbind(betax*FFx3+gammax*FFy3, betay*FFy3+gammay*FFx3)+mvrnorm(NN,rep(0,2), matrix(c(omegax,omegaxy,omegaxy,omegay),2,2));
FFx4<-FF4[,1]; FFy4<-FF4[,2]
DT4<-FF4+c1*mvrnorm(NN, rep(0,2),matrix(c(psix2,psixy,psixy,psiy2),2,2))+(c(mux4,muy4)+ c2*cbind(Tx,Ty))
FF5<- cbind(betax*FFx4+gammax*FFy4, betay*FFy4+gammay*FFx4)+mvrnorm(NN,rep(0,2), matrix(c(omegax,omegaxy,omegaxy,omegay),2,2));
FFx5<-FF5[,1]; FFy5<-FF5[,2]
DT5<-FF5+c1*mvrnorm(NN, rep(0,2),matrix(c(psix2,psixy,psixy,psiy2),2,2))+(c(mux5,muy5)+ c2*cbind(Tx,Ty))
FF6<- cbind(betax*FFx5+gammax*FFy5, betay*FFy5+gammay*FFx5)+mvrnorm(NN,rep(0,2), matrix(c(omegax,omegaxy,omegaxy,omegay),2,2));
FFx6<-FF6[,1]; FFy6<-FF6[,2]
DT6<-FF6+c1*mvrnorm(NN, rep(0,2),matrix(c(psix2,psixy,psixy,psiy2),2,2))+(c(mux6,muy6)+ c2*cbind(Tx,Ty))
FF7<- cbind(betax*FFx6+gammax*FFy6, betay*FFy6+gammay*FFx6)+mvrnorm(NN,rep(0,2), matrix(c(omegax,omegaxy,omegaxy,omegay),2,2));
FFx7<-FF7[,1]; FFy7<-FF7[,2]
DT7<-FF7+c1*mvrnorm(NN, rep(0,2),matrix(c(psix2,psixy,psixy,psiy2),2,2))+(c(mux7,muy7)+ c2*cbind(Tx,Ty))
FF8<- cbind(betax*FFx7+gammax*FFy7, betay*FFy7+gammay*FFx7)+mvrnorm(NN,rep(0,2), matrix(c(omegax,omegaxy,omegaxy,omegay),2,2));
FFx8<-FF8[,1]; FFy8<-FF8[,2]
DT8<-FF8+c1*mvrnorm(NN, rep(0,2),matrix(c(psix2,psixy,psixy,psiy2),2,2))+(c(mux8,muy8)+ c2*cbind(Tx,Ty))

Data<-data.frame(cbind(DT1,DT2,DT3,DT4,DT5,DT6,DT7,DT8))
colnames(Data)<-c("x1","y1","x2","y2","x3","y3","x4","y4","x5","y5","x6","y6","x7","y7","x8","y8")
```

```
DATA<-Data[,1:(2*TT)]

#DATA analysis to evaluate Bias, SE and statistical significance.

fit<- suppressMessages(suppressWarnings (lavaan(CLPMT4, data = DATA,control=list(iter.max=50)))) #change the red-highlighted part for different T
COU1<-sign(sum(diag(inspect(fit, "coefficients")$psi)<0)+sum(diag(inspect(fit, "coefficients")$theta)<0)+sum(det(fitted(fit)$cov)<0))
CLGSD<- parameterEstimates(fit,standardized=TRUE)[2*TT+9,12];CLG<-inspect(fit, "coefficients")$beta[2+TT,1]
CLAIC<- AIC(fit); CLBIC<-BIC(fit);CLGbias<-inspect(fit, "coefficients")$beta[2+TT,1]-gammax;CLGse<- parameterEstimates(fit,standardized=TRUE)[2*TT+9,6]
SIGCLG<- (sign(0.05-parameterEstimates(fit)[2*TT+9,8])+1)/2

fit<- suppressMessages(suppressWarnings (lavaan(RICLPMT4, data = DATA,control=list(iter.max=50))))#change the red-highlighted part for different T
COU2<-sign(sum(diag(inspect(fit, "coefficients")$psi)<0)+sum(diag(inspect(fit, "coefficients")$theta)<0)+sum(det(fitted(fit)$cov)<0))
RICLGSD<- parameterEstimates(fit,standardized=TRUE)[4*TT+14,12];RICLG<-inspect(fit, "coefficients")$beta[4+TT,3]
RICLAIC<-                    AIC(fit);                    RICLBIC<-BIC(fit);RICLGbias<-inspect(fit,                    "coefficients")$beta[4+TT,3]-gammax;RICLGse<-
parameterEstimates(fit,standardized=TRUE)[4*TT+14,6]
SIGRICLG<- (sign(0.05-parameterEstimates(fit)[4*TT+14,8])+1)/2

fit<- suppressMessages(suppressWarnings(lavaan(STARTST4, data = DATA,control=list(iter.max=50))))#change the red-highlighted part for different T
COU3<-sign(sum(diag(inspect(fit, "coefficients")$psi)<0)+sum(diag(inspect(fit, "coefficients")$theta)<0)+sum(det(fitted(fit)$cov)<0))
STGSD<- parameterEstimates(fit, standardized=TRUE)[7*TT+14,12];STG<-inspect(fit, "coefficients")$beta[4+TT,3]
STAIC<-AIC(fit);STBIC<-BIC(fit);STGbias<-inspect(fit, "coefficients")$beta[4+TT,3]-gammax; STGse<- parameterEstimates(fit,standardized=TRUE)[7*TT+14,6]
SIGSTG<- (sign(0.05-parameterEstimates(fit)[7*TT+14,8])+1)/2

SIGCLRICLtype<-2*SIGCLG+(SIGRICLG+1)
SIGCLSTtype<-2*SIGCLG+(SIGSTG+1)
SIGRICLSTtype<-2*SIGRICLG+(SIGSTG+1)

if((1-COU1)*(1-COU2)*(1-COU3)>0){
AIC1ES<-AIC1ES+ifelse(rank(c(CLAIC,RICLAIC,STAIC))[1]==1,1,0)
BIC1ES<- BIC1ES+ifelse(rank(c(CLBIC,RICLBIC,STBIC))[1]==1,1,0)
AIC2ES<-AIC2ES+ifelse(rank(c(CLAIC,RICLAIC,STAIC))[2]==1,1,0)
BIC2ES<- BIC2ES+ifelse(rank(c(CLBIC,RICLBIC,STBIC))[2]==1,1,0)
AIC3ES<-AIC3ES+ifelse(rank(c(CLAIC,RICLAIC,STAIC))[3]==1,1,0)
BIC3ES<- BIC3ES+ifelse(rank(c(CLBIC,RICLBIC,STBIC))[3]==1,1,0)
ttt<-ttt+1
CLGSDES[ttt]<-CLGSD
CLGES[ttt]<-CLG
RICLGSDES[ttt]<-RICLGSD
RICLGES[ttt]<-RICLG
STGSDES[ttt]<-STGSD
STGES[ttt]<-STG
CLGESbias[ttt]<-CLGbias
CLGESse[ttt]<-CLGse
RICLGESbias[ttt]<-RICLGbias
RICLGESse[ttt]<-RICLGse
STGESbias[ttt]<-STGbias
STGESse[ttt]<-STGse
sumSIGDiffCLRICLG1<- sumSIGDiffCLRICLG1+ifelse(SIGCLRICLtype==1,1,0)
sumSIGDiffCLRICLG2<- sumSIGDiffCLRICLG2+ifelse(SIGCLRICLtype==2,1,0)
sumSIGDiffCLRICLG3<- sumSIGDiffCLRICLG3+ifelse(SIGCLRICLtype==3,1,0)
sumSIGDiffCLRICLG4<- sumSIGDiffCLRICLG4+ifelse(SIGCLRICLtype==4,1,0)
sumSIGDiffCLSTG1<- sumSIGDiffCLSTG1+ifelse(SIGCLSTtype==1,1,0)
sumSIGDiffCLSTG2<- sumSIGDiffCLSTG2+ifelse(SIGCLSTtype==2,1,0)
sumSIGDiffCLSTG3<- sumSIGDiffCLSTG3+ifelse(SIGCLSTtype==3,1,0)
sumSIGDiffCLSTG4<- sumSIGDiffCLSTG4+ifelse(SIGCLSTtype==4,1,0)
sumSIGDiffRICLSTG1<- sumSIGDiffRICLSTG1+ifelse(SIGRICLSTtype==1,1,0)
sumSIGDiffRICLSTG2<- sumSIGDiffRICLSTG2+ifelse(SIGRICLSTtype==2,1,0)
sumSIGDiffRICLSTG3<- sumSIGDiffRICLSTG3+ifelse(SIGRICLSTtype==3,1,0)
sumSIGDiffRICLSTG4<- sumSIGDiffRICLSTG4+ifelse(SIGRICLSTtype==4,1,0)
}else{
}

}

  RESULT[3*2*3*(aaaa-1)+2*3*(cccc-1)+3*(bbbb-1)+dddd,1]<-mean(CLGSDES)
  RESULT[3*2*3*(aaaa-1)+2*3*(cccc-1)+3*(bbbb-1)+dddd,2]<-mean(RICLGSDES)
  RESULT[3*2*3*(aaaa-1)+2*3*(cccc-1)+3*(bbbb-1)+dddd,3]<-mean(STGSDES)
  RESULT[3*2*3*(aaaa-1)+2*3*(cccc-1)+3*(bbbb-1)+dddd,4]<-mean(CLGES)
  RESULT[3*2*3*(aaaa-1)+2*3*(cccc-1)+3*(bbbb-1)+dddd,5]<-mean(RICLGES)
  RESULT[3*2*3*(aaaa-1)+2*3*(cccc-1)+3*(bbbb-1)+dddd,6]<-mean(STGES)
  RESULT[3*2*3*(aaaa-1)+2*3*(cccc-1)+3*(bbbb-1)+dddd,7]<-mean(CLGESse)
  RESULT[3*2*3*(aaaa-1)+2*3*(cccc-1)+3*(bbbb-1)+dddd,8]<-mean(RICLGESse)
  RESULT[3*2*3*(aaaa-1)+2*3*(cccc-1)+3*(bbbb-1)+dddd,9]<-mean(STGESse)
RESULT[3*2*3*(aaaa-1)+2*3*(cccc-1)+3*(bbbb-1)+dddd,10] <-mean(CLGESbias)
  RESULT[3*2*3*(aaaa-1)+2*3*(cccc-1)+3*(bbbb-1)+dddd,11] <-mean(RICLGESbias)
  RESULT[3*2*3*(aaaa-1)+2*3*(cccc-1)+3*(bbbb-1)+dddd,12] <- mean(STGESbias)
RESULT[3*2*3*(aaaa-1)+2*3*(cccc-1)+3*(bbbb-1)+dddd,13] <-AIC1ES
RESULT[3*2*3*(aaaa-1)+2*3*(cccc-1)+3*(bbbb-1)+dddd,14] <-AIC2ES
RESULT[3*2*3*(aaaa-1)+2*3*(cccc-1)+3*(bbbb-1)+dddd,15] <-AIC3ES
RESULT[3*2*3*(aaaa-1)+2*3*(cccc-1)+3*(bbbb-1)+dddd,16] <-BIC1ES
RESULT[3*2*3*(aaaa-1)+2*3*(cccc-1)+3*(bbbb-1)+dddd,17] <-BIC2ES
RESULT[3*2*3*(aaaa-1)+2*3*(cccc-1)+3*(bbbb-1)+dddd,18] <-BIC3ES
RESULT[3*2*3*(aaaa-1)+2*3*(cccc-1)+3*(bbbb-1)+dddd,19] <- sd(CLGESbias)
  RESULT[3*2*3*(aaaa-1)+2*3*(cccc-1)+3*(bbbb-1)+dddd,20] <- sd(RICLGESbias)
  RESULT[3*2*3*(aaaa-1)+2*3*(cccc-1)+3*(bbbb-1)+dddd,21] <- sd(STGESbias)
  RESULT[3*2*3*(aaaa-1)+2*3*(cccc-1)+3*(bbbb-1)+dddd,22]<-mean(CLGES-RICLGES)
  RESULT[3*2*3*(aaaa-1)+2*3*(cccc-1)+3*(bbbb-1)+dddd,23] <-mean(CLGES-STGES)
  RESULT[3*2*3*(aaaa-1)+2*3*(cccc-1)+3*(bbbb-1)+dddd,24] <-mean(RICLGES-STGES)
  RESULT[3*2*3*(aaaa-1)+2*3*(cccc-1)+3*(bbbb-1)+dddd,25]<-sd(CLGES-RICLGES)
  RESULT[3*2*3*(aaaa-1)+2*3*(cccc-1)+3*(bbbb-1)+dddd,26] <-sd(CLGES-STGES)
  RESULT[3*2*3*(aaaa-1)+2*3*(cccc-1)+3*(bbbb-1)+dddd,27] <-sd(RICLGES-STGES)
  RESULT[3*2*3*(aaaa-1)+2*3*(cccc-1)+3*(bbbb-1)+dddd,28]<-sumSIGDiffCLRICLG1/Repeat
  RESULT[3*2*3*(aaaa-1)+2*3*(cccc-1)+3*(bbbb-1)+dddd,29]<-sumSIGDiffCLRICLG2/Repeat
  RESULT[3*2*3*(aaaa-1)+2*3*(cccc-1)+3*(bbbb-1)+dddd,30]<-sumSIGDiffCLRICLG3/Repeat
  RESULT[3*2*3*(aaaa-1)+2*3*(cccc-1)+3*(bbbb-1)+dddd,31]<-sumSIGDiffCLRICLG4/Repeat
  RESULT[3*2*3*(aaaa-1)+2*3*(cccc-1)+3*(bbbb-1)+dddd,32]<-sumSIGDiffCLSTG1/Repeat
  RESULT[3*2*3*(aaaa-1)+2*3*(cccc-1)+3*(bbbb-1)+dddd,33]<-sumSIGDiffCLSTG2/Repeat
  RESULT[3*2*3*(aaaa-1)+2*3*(cccc-1)+3*(bbbb-1)+dddd,34]<-sumSIGDiffCLSTG3/Repeat
  RESULT[3*2*3*(aaaa-1)+2*3*(cccc-1)+3*(bbbb-1)+dddd,35]<-sumSIGDiffCLSTG4/Repeat
  RESULT[3*2*3*(aaaa-1)+2*3*(cccc-1)+3*(bbbb-1)+dddd,36]<-sumSIGDiffRICLSTG1/Repeat
  RESULT[3*2*3*(aaaa-1)+2*3*(cccc-1)+3*(bbbb-1)+dddd,37]<-sumSIGDiffRICLSTG2/Repeat
  RESULT[3*2*3*(aaaa-1)+2*3*(cccc-1)+3*(bbbb-1)+dddd,38]<-sumSIGDiffRICLSTG3/Repeat
  RESULT[3*2*3*(aaaa-1)+2*3*(cccc-1)+3*(bbbb-1)+dddd,39]<-sumSIGDiffRICLSTG4/Repeat
RESULT[3*2*3*(aaaa-1)+2*3*(cccc-1)+3*(bbbb-1)+dddd,40]<-gammax
RESULT[3*2*3*(aaaa-1)+2*3*(cccc-1)+3*(bbbb-1)+dddd,41]<-betax
RESULT[3*2*3*(aaaa-1)+2*3*(cccc-1)+3*(bbbb-1)+dddd,42]<-psix2
RESULT[3*2*3*(aaaa-1)+2*3*(cccc-1)+3*(bbbb-1)+dddd,43]<-NN
};};};}
write.csv(RESULT,"Specifying an appropriate directory and file name here")
}
```

#Simulation Code when the RI-CLPM is the data generation model (T=4)

```
SIMULATIONRICLPMT4<-function(Repeat){
library(MASS) ;require("lavaan");RESULT<-matrix(rep(0,3**3*2*43),3**3*2,43)
options(warn=-1)
for(aaaa in 1:3){
for(bbbb in 1:2){
for(cccc in 1:3){
for(dddd in 1:3){

TT<-4;c1<-0;c2<-1

NN<-c(200,600,1000)[dddd]
#Specifying parameters
psix2<-c(0.2,0.4,0.6)[cccc];psiy2<-c(0.2,0.4,0.6)[cccc];psixy<-0.2*psix2 #measurement error (co)variances
phix12<-1-psix2; phiy12<-1-psiy2;phix1y1<-0.2*phix12 #phi1 is the variance at the first time point
phiTx2<-phix12*0.5; phiTy2<-phiy12*0.5;phiTxTy<-0.2*phiTx2    #specifying the trait factor (co)variances
phix1Tx<-0.1*sqrt(phix12*phiTx2);phix1Ty<-0.1*sqrt(phix12*phiTy2) #specifying correlations
phiy1Tx<-0.1*sqrt(phiy12*phiTx2);phiy1Ty<-0.1*sqrt(phiy12*phiTy2) #specifying correlations
betax<-c(0.5,0.7)[bbbb];    betay<-c(0.5,0.7)[bbbb]; gammax<-c(0.0,0.1,0.2)[aaaa];gammay<- c(0.0,0.1,0.2)[aaaa] #autoregressive and cross-lagged parameters
omegax<-0.2; omegay<-0.2; omegaxy<-0.2*omegax    #residual (co)variances
mux1<-0; mux2<-1; mux3<-2; mux4<-3; mux5<-4; mux6<-5; mux7<-6; mux8<-7 #temporal group means
muy1<-0; muy2<-1; muy3<-2; muy4<-3; muy5<-4; muy6<-5; muy7<-6; muy8<-7
muTx<-0; muTy<-0 #trait factor means (=0)

ttt<-0
CLGSDES<-rep(0,Repeat);CLGES<- rep(0,Repeat);RICLGSDES<- rep(0,Repeat);RICLGES<- rep(0,Repeat);STGSDES<- rep(0,Repeat);STGES<- rep(0,Repeat)
CLGESbias<- rep(0,Repeat); RICLGESbias<- rep(0,Repeat);STGESbias<- rep(0,Repeat)
CLGESse<- rep(0,Repeat); RICLGESse<- rep(0,Repeat);STGESse<- rep(0,Repeat)
AIC1ES<- 0; AIC2ES<-0;AIC3ES<- 0; BIC1ES<- 0; BIC2ES<- 0;BIC3ES<- 0

sumSIGDiffCLRICLG1<- 0;sumSIGDiffCLRICLG2<- 0;sumSIGDiffCLRICLG3<- 0;sumSIGDiffCLRICLG4<-0
sumSIGDiffCLSTG1<- 0;sumSIGDiffCLSTG2<-0;sumSIGDiffCLSTG3<- 0;sumSIGDiffCLSTG4<-0
sumSIGDiffRICLSTG1<- 0;sumSIGDiffRICLSTG2<- 0;sumSIGDiffRICLSTG3<- 0;sumSIGDiffRICLSTG4<- 0
while(ttt<Repeat){
Factors<-mvrnorm(NN,c(mux1,muy1,muTx,muTy),
matrix(c(
phix12,phix1y1,phix1Tx,phix1Ty,
phix1y1,phiy12,phiy1Tx,phiy1Ty,
phix1Tx,phiy1Tx,phiTx2,phiTxTy,
phix1Ty,phiy1Ty,phiTxTy,phiTy2
),4,4))

#DATA generation
FFx1<-Factors[,1]; FFy1<-Factors[,2]; Tx<-Factors[,3]; Ty<-Factors[,4];
DT1<-cbind(FFx1,FFy1)+c1*mvrnorm(NN, rep(0,2),matrix(c(psix2,psixy,psixy,psiy2),2,2))+(c(mux1,muy1)+ c2*cbind(Tx,Ty))
FF2<- cbind(betax*FFx1+gammax*FFy1, betay*FFy1+gammay*FFx1)+mvrnorm(NN,rep(0,2), matrix(c(omegax,omegaxy,omegaxy,omegay),2,2));
FFx2<-FF2[,1]; FFy2<-FF2[,2]
DT2<-FF2+c1*mvrnorm(NN, rep(0,2),matrix(c(psix2,psixy,psixy,psiy2),2,2))+(c(mux2,muy2)+ c2*cbind(Tx,Ty))
FF3<- cbind(betax*FFx2+gammax*FFy2, betay*FFy2+gammay*FFx2)+mvrnorm(NN,rep(0,2), matrix(c(omegax,omegaxy,omegaxy,omegay),2,2));
FFx3<-FF3[,1]; FFy3<-FF3[,2]
DT3<-FF3+c1*mvrnorm(NN, rep(0,2),matrix(c(psix2,psixy,psixy,psiy2),2,2))+(c(mux3,muy3)+ c2*cbind(Tx,Ty))
FF4<- cbind(betax*FFx3+gammax*FFy3, betay*FFy3+gammay*FFx3)+mvrnorm(NN,rep(0,2), matrix(c(omegax,omegaxy,omegaxy,omegay),2,2));
FFx4<-FF4[,1]; FFy4<-FF4[,2]
DT4<-FF4+c1*mvrnorm(NN, rep(0,2),matrix(c(psix2,psixy,psixy,psiy2),2,2))+(c(mux4,muy4)+ c2*cbind(Tx,Ty))
FF5<- cbind(betax*FFx4+gammax*FFy4, betay*FFy4+gammay*FFx4)+mvrnorm(NN,rep(0,2), matrix(c(omegax,omegaxy,omegaxy,omegay),2,2));
FFx5<-FF5[,1]; FFy5<-FF5[,2]
DT5<-FF5+c1*mvrnorm(NN, rep(0,2),matrix(c(psix2,psixy,psixy,psiy2),2,2))+(c(mux5,muy5)+ c2*cbind(Tx,Ty))
FF6<- cbind(betax*FFx5+gammax*FFy5, betay*FFy5+gammay*FFx5)+mvrnorm(NN,rep(0,2), matrix(c(omegax,omegaxy,omegaxy,omegay),2,2));
FFx6<-FF6[,1]; FFy6<-FF6[,2]
DT6<-FF6+c1*mvrnorm(NN, rep(0,2),matrix(c(psix2,psixy,psixy,psiy2),2,2))+(c(mux6,muy6)+ c2*cbind(Tx,Ty))
FF7<- cbind(betax*FFx6+gammax*FFy6, betay*FFy6+gammay*FFx6)+mvrnorm(NN,rep(0,2), matrix(c(omegax,omegaxy,omegaxy,omegay),2,2));
FFx7<-FF7[,1]; FFy7<-FF7[,2]
DT7<-FF7+c1*mvrnorm(NN, rep(0,2),matrix(c(psix2,psixy,psixy,psiy2),2,2))+(c(mux7,muy7)+ c2*cbind(Tx,Ty))
FF8<- cbind(betax*FFx7+gammax*FFy7, betay*FFy7+gammay*FFx7)+mvrnorm(NN,rep(0,2), matrix(c(omegax,omegaxy,omegaxy,omegay),2,2));
FFx8<-FF8[,1]; FFy8<-FF8[,2]
DT8<-FF8+c1*mvrnorm(NN, rep(0,2),matrix(c(psix2,psixy,psixy,psiy2),2,2))+(c(mux8,muy8)+ c2*cbind(Tx,Ty))

Data<-data.frame(cbind(DT1,DT2,DT3,DT4,DT5,DT6,DT7,DT8))
colnames(Data)<-c("x1","y1","x2","y2","x3","y3","x4","y4","x5","y5","x6","y6","x7","y7","x8","y8")
DATA<-Data[,1:(2*TT)]
```

#DATA analysis to evaluate Bias, SE and statistical significance.

```
fit<- suppressMessages(suppressWarnings (lavaan(CLPMT4, data = DATA,control=list(iter.max=50)))) #change the red-highlighted part for different T
COU1<-sign(sum(diag(inspect(fit, "coefficients")$psi)<0)+sum(diag(inspect(fit, "coefficients")$theta)<0)+sum(det(fitted(fit)$cov)<0))
CLGSD<- parameterEstimates(fit,standardized=TRUE)[2*TT+9,12];CLG<-inspect(fit, "coefficients")$beta[2+TT,1]
CLAIC<- AIC(fit); CLBIC<-BIC(fit);CLGbias<-inspect(fit, "coefficients")$beta[2+TT,1]-gammax;CLGse<- parameterEstimates(fit,standardized=TRUE)[2*TT+9,6]
SIGCLG<- (sign(0.05-parameterEstimates(fit)[2*TT+9,8])+1)/2

fit<- suppressMessages(suppressWarnings (lavaan(RICLPMT4, data = DATA,control=list(iter.max=50))))#change the red-highlighted part for different T
COU2<-sign(sum(diag(inspect(fit, "coefficients")$psi)<0)+sum(diag(inspect(fit, "coefficients")$theta)<0)+sum(det(fitted(fit)$cov)<0))
RICLGSD<- parameterEstimates(fit,standardized=TRUE)[4*TT+14,12];RICLG<-inspect(fit, "coefficients")$beta[4+TT,3]
RICLAIC<- AIC(fit); RICLBIC<-BIC(fit);RICLGbias<-inspect(fit, "coefficients")$beta[4+TT,3]-gammax;RICLGse<-
parameterEstimates(fit,standardized=TRUE)[4*TT+14,6]
SIGRICLG<- (sign(0.05-parameterEstimates(fit)[4*TT+14,8])+1)/2

fit<- suppressMessages(suppressWarnings(lavaan(STARTST4, data = DATA,control=list(iter.max=50))))#change the red-highlighted part for different T
COU3<-sign(sum(diag(inspect(fit, "coefficients")$psi)<0)+sum(diag(inspect(fit, "coefficients")$theta)<0)+sum(det(fitted(fit)$cov)<0))
STGSD<- parameterEstimates(fit, standardized=TRUE)[7*TT+14,12];STG<-inspect(fit, "coefficients")$beta[4+TT,3]
STAIC<-AIC(fit);STBIC<-BIC(fit);STGbias<-inspect(fit, "coefficients")$beta[4+TT,3]-gammax; STGse<- parameterEstimates(fit,standardized=TRUE)[7*TT+14,6]
SIGSTG<- (sign(0.05-parameterEstimates(fit)[7*TT+14,8])+1)/2

SIGCLRICLtype<-2*SIGCLG+(SIGRICLG+1)
SIGCLSTtype<-2*SIGCLG+(SIGSTG+1)
SIGRICLSTtype<-2*SIGRICLG+(SIGSTG+1)

if((1-COU1)*(1-COU2)*(1-COU3)>0){
AIC1ES<-AIC1ES+ifelse(rank(c(CLAIC,RICLAIC,STAIC))[1]==1,1,0)
BIC1ES<- BIC1ES+ifelse(rank(c(CLBIC,RICLBIC,STBIC))[1]==1,1,0)
AIC2ES<-AIC2ES+ifelse(rank(c(CLAIC,RICLAIC,STAIC))[2]==1,1,0)
BIC2ES<- BIC2ES+ifelse(rank(c(CLBIC,RICLBIC,STBIC))[2]==1,1,0)
AIC3ES<-AIC3ES+ifelse(rank(c(CLAIC,RICLAIC,STAIC))[3]==1,1,0)
BIC3ES<- BIC3ES+ifelse(rank(c(CLBIC,RICLBIC,STBIC))[3]==1,1,0)
ttt<-ttt+1
CLGSDES[ttt]<-CLGSD
CLGES[ttt]<-CLG
RICLGSDES[ttt]<-RICLGSD
RICLGES[ttt]<-RICLG
STGSDES[ttt]<-STGSD
STGES[ttt]<-STG
CLGESbias[ttt]<-CLGbias
CLGESse[ttt]<-CLGse
RICLGESbias[ttt]<-RICLGbias
RICLGESse[ttt]<-RICLGse
STGESbias[ttt]<-STGbias
STGESse[ttt]<-STGse
sumSIGDiffCLRICLG1<- sumSIGDiffCLRICLG1+ifelse(SIGCLRICLtype==1,1,0)
sumSIGDiffCLRICLG2<- sumSIGDiffCLRICLG2+ifelse(SIGCLRICLtype==2,1,0)
sumSIGDiffCLRICLG3<- sumSIGDiffCLRICLG3+ifelse(SIGCLRICLtype==3,1,0)
sumSIGDiffCLRICLG4<- sumSIGDiffCLRICLG4+ifelse(SIGCLRICLtype==4,1,0)
sumSIGDiffCLSTG1<- sumSIGDiffCLSTG1+ifelse(SIGCLSTtype==1,1,0)
sumSIGDiffCLSTG2<- sumSIGDiffCLSTG2+ifelse(SIGCLSTtype==2,1,0)
sumSIGDiffCLSTG3<- sumSIGDiffCLSTG3+ifelse(SIGCLSTtype==3,1,0)
sumSIGDiffCLSTG4<- sumSIGDiffCLSTG4+ifelse(SIGCLSTtype==4,1,0)
sumSIGDiffRICLSTG1<- sumSIGDiffRICLSTG1+ifelse(SIGRICLSTtype==1,1,0)
sumSIGDiffRICLSTG2<- sumSIGDiffRICLSTG2+ifelse(SIGRICLSTtype==2,1,0)
sumSIGDiffRICLSTG3<- sumSIGDiffRICLSTG3+ifelse(SIGRICLSTtype==3,1,0)
sumSIGDiffRICLSTG4<- sumSIGDiffRICLSTG4+ifelse(SIGRICLSTtype==4,1,0)
}else{
}

}

  RESULT[3*2*3*(aaaa-1)+2*3*(cccc-1)+3*(bbbb-1)+dddd,1]<-mean(CLGSDES)
  RESULT[3*2*3*(aaaa-1)+2*3*(cccc-1)+3*(bbbb-1)+dddd,2]<-mean(RICLGSDES)
  RESULT[3*2*3*(aaaa-1)+2*3*(cccc-1)+3*(bbbb-1)+dddd,3]<-mean(STGSDES)
  RESULT[3*2*3*(aaaa-1)+2*3*(cccc-1)+3*(bbbb-1)+dddd,4]<-mean(CLGES)
  RESULT[3*2*3*(aaaa-1)+2*3*(cccc-1)+3*(bbbb-1)+dddd,5]<-mean(RICLGES)
  RESULT[3*2*3*(aaaa-1)+2*3*(cccc-1)+3*(bbbb-1)+dddd,6]<-mean(STGES)
  RESULT[3*2*3*(aaaa-1)+2*3*(cccc-1)+3*(bbbb-1)+dddd,7]<-mean(CLGESse)
  RESULT[3*2*3*(aaaa-1)+2*3*(cccc-1)+3*(bbbb-1)+dddd,8]<-mean(RICLGESse)
  RESULT[3*2*3*(aaaa-1)+2*3*(cccc-1)+3*(bbbb-1)+dddd,9]<-mean(STGESse)
RESULT[3*2*3*(aaaa-1)+2*3*(cccc-1)+3*(bbbb-1)+dddd,10] <-mean(CLGESbias)
  RESULT[3*2*3*(aaaa-1)+2*3*(cccc-1)+3*(bbbb-1)+dddd,11] <-mean(RICLGESbias)
  RESULT[3*2*3*(aaaa-1)+2*3*(cccc-1)+3*(bbbb-1)+dddd,12] <- mean(STGESbias)
RESULT[3*2*3*(aaaa-1)+2*3*(cccc-1)+3*(bbbb-1)+dddd,13] <-AIC1ES
RESULT[3*2*3*(aaaa-1)+2*3*(cccc-1)+3*(bbbb-1)+dddd,14] <-AIC2ES
RESULT[3*2*3*(aaaa-1)+2*3*(cccc-1)+3*(bbbb-1)+dddd,15] <-AIC3ES
RESULT[3*2*3*(aaaa-1)+2*3*(cccc-1)+3*(bbbb-1)+dddd,16] <-BIC1ES
RESULT[3*2*3*(aaaa-1)+2*3*(cccc-1)+3*(bbbb-1)+dddd,17] <-BIC2ES
RESULT[3*2*3*(aaaa-1)+2*3*(cccc-1)+3*(bbbb-1)+dddd,18] <-BIC3ES
RESULT[3*2*3*(aaaa-1)+2*3*(cccc-1)+3*(bbbb-1)+dddd,19] <- sd(CLGESbias)
  RESULT[3*2*3*(aaaa-1)+2*3*(cccc-1)+3*(bbbb-1)+dddd,20] <- sd(RICLGESbias)
  RESULT[3*2*3*(aaaa-1)+2*3*(cccc-1)+3*(bbbb-1)+dddd,21] <- sd(STGESbias)
  RESULT[3*2*3*(aaaa-1)+2*3*(cccc-1)+3*(bbbb-1)+dddd,22]<-mean(CLGES-RICLGES)
  RESULT[3*2*3*(aaaa-1)+2*3*(cccc-1)+3*(bbbb-1)+dddd,23] <-mean(CLGES-STGES)
  RESULT[3*2*3*(aaaa-1)+2*3*(cccc-1)+3*(bbbb-1)+dddd,24] <-mean(RICLGES-STGES)
  RESULT[3*2*3*(aaaa-1)+2*3*(cccc-1)+3*(bbbb-1)+dddd,25]<-sd(CLGES-RICLGES)
  RESULT[3*2*3*(aaaa-1)+2*3*(cccc-1)+3*(bbbb-1)+dddd,26] <-sd(CLGES-STGES)
  RESULT[3*2*3*(aaaa-1)+2*3*(cccc-1)+3*(bbbb-1)+dddd,27] <-sd(RICLGES-STGES)
  RESULT[3*2*3*(aaaa-1)+2*3*(cccc-1)+3*(bbbb-1)+dddd,28]<-sumSIGDiffCLRICLG1/Repeat
  RESULT[3*2*3*(aaaa-1)+2*3*(cccc-1)+3*(bbbb-1)+dddd,29]<-sumSIGDiffCLRICLG2/Repeat
  RESULT[3*2*3*(aaaa-1)+2*3*(cccc-1)+3*(bbbb-1)+dddd,30]<-sumSIGDiffCLRICLG3/Repeat
  RESULT[3*2*3*(aaaa-1)+2*3*(cccc-1)+3*(bbbb-1)+dddd,31]<-sumSIGDiffCLRICLG4/Repeat
  RESULT[3*2*3*(aaaa-1)+2*3*(cccc-1)+3*(bbbb-1)+dddd,32]<-sumSIGDiffCLSTG1/Repeat
  RESULT[3*2*3*(aaaa-1)+2*3*(cccc-1)+3*(bbbb-1)+dddd,33]<-sumSIGDiffCLSTG2/Repeat
  RESULT[3*2*3*(aaaa-1)+2*3*(cccc-1)+3*(bbbb-1)+dddd,34]<-sumSIGDiffCLSTG3/Repeat
  RESULT[3*2*3*(aaaa-1)+2*3*(cccc-1)+3*(bbbb-1)+dddd,35]<-sumSIGDiffCLSTG4/Repeat
  RESULT[3*2*3*(aaaa-1)+2*3*(cccc-1)+3*(bbbb-1)+dddd,36]<-sumSIGDiffRICLSTG1/Repeat
  RESULT[3*2*3*(aaaa-1)+2*3*(cccc-1)+3*(bbbb-1)+dddd,37]<-sumSIGDiffRICLSTG2/Repeat
  RESULT[3*2*3*(aaaa-1)+2*3*(cccc-1)+3*(bbbb-1)+dddd,38]<-sumSIGDiffRICLSTG3/Repeat
  RESULT[3*2*3*(aaaa-1)+2*3*(cccc-1)+3*(bbbb-1)+dddd,39]<-sumSIGDiffRICLSTG4/Repeat
RESULT[3*2*3*(aaaa-1)+2*3*(cccc-1)+3*(bbbb-1)+dddd,40]<-gammax
RESULT[3*2*3*(aaaa-1)+2*3*(cccc-1)+3*(bbbb-1)+dddd,41]<-betax
RESULT[3*2*3*(aaaa-1)+2*3*(cccc-1)+3*(bbbb-1)+dddd,42]<-psix2
RESULT[3*2*3*(aaaa-1)+2*3*(cccc-1)+3*(bbbb-1)+dddd,43]<-NN
};};};}
write.csv(RESULT,"Specifying an appropriate directory and file name here")
}
```

#Simulation Code when the STARTS model is the data generation model (T=4)

```
SIMULATIONSTARTST4<-function(Repeat){
library(MASS) ;require("lavaan");RESULT<-matrix(rep(0,3**3*2*43),3**3*2,43)
options(warn=-1)
for(aaaa in 1:3){
for(bbbb in 1:2){
for(cccc in 1:3){
for(dddd in 1:3){

TT<-4;c1<-1;c2<-1;c3<-0; c4<-0;alpha<-1

NN<-c(200,600,1000)[dddd]
```

#Specifying parameters

```
psix2<-c(0.2,0.4,0.6)[cccc];psiy2<-c(0.2,0.4,0.6)[cccc];psixy<-0.2*psix2 #measurement error (co)variances
phix12<-1-psix2; phiy12<-1-psiy2;phix1y1<-0.2*phix12 #phi1 is the variance at the first time point
phiTx2<-phix12*0.5; phiTy2<-phiy12*0.5;phiTxTy<-0.2*phiTx2    #specifying the trait factor (co)variances
phix1Tx<-0.1*sqrt(phix12*phiTx2);phix1Ty<-0.1*sqrt(phix12*phiTy2) #specifying correlations
phiy1Tx<-0.1*sqrt(phiy12*phiTx2);phiy1Ty<-0.1*sqrt(phiy12*phiTy2) #specifying correlations
betax<-c(0.5,0.7)[bbbb];    betay<-c(0.5,0.7)[bbbb]; gammax<-c(0.0,0.1,0.2)[aaaa];gammay<- c(0.0,0.1,0.2)[aaaa] #autoregressive and cross-lagged parameters
omegax<-0.2; omegay<-0.2; omegaxy<-0.2*omegax    #residual (co)variances
mux1<-0; mux2<-1; mux3<-2; mux4<-3; mux5<-4; mux6<-5; mux7<-6; mux8<-7 #temporal group means
muy1<-0; muy2<-1; muy3<-2; muy4<-3; muy5<-4; muy6<-5; muy7<-6; muy8<-7
muTx<-0; muTy<-0 #trait factor means (=0)

ttt<-0
CLGSDES<-rep(0,Repeat);CLGES<- rep(0,Repeat);RICLGSDES<- rep(0,Repeat);RICLGES<- rep(0,Repeat);STGSDES<- rep(0,Repeat);STGES<- rep(0,Repeat)
CLGESbias<- rep(0,Repeat); RICLGESbias<- rep(0,Repeat);STGESbias<- rep(0,Repeat)
CLGESse<- rep(0,Repeat); RICLGESse<- rep(0,Repeat);STGESse<- rep(0,Repeat)
AIC1ES<- 0; AIC2ES<-0;AIC3ES<- 0; BIC1ES<- 0; BIC2ES<- 0;BIC3ES<- 0

sumSIGDiffCLRICLG1<- 0;sumSIGDiffCLRICLG2<- 0;sumSIGDiffCLRICLG3<- 0;sumSIGDiffCLRICLG4<-0
sumSIGDiffCLSTG1<- 0;sumSIGDiffCLSTG2<-0;sumSIGDiffCLSTG3<- 0;sumSIGDiffCLSTG4<-0
sumSIGDiffRICLSTG1<- 0;sumSIGDiffRICLSTG2<- 0;sumSIGDiffRICLSTG3<- 0;sumSIGDiffRICLSTG4<- 0
while(ttt<Repeat){
Factors<-mvrnorm(NN,c(mux1,muy1,muTx,muTy),
matrix(c(
phix12,phix1y1,phix1Tx,phix1Ty,
phix1y1,phiy12,phiy1Tx,phiy1Ty,
phix1Tx,phiy1Tx,phiTx2,phiTxTy,
phix1Ty,phiy1Ty,phiTxTy,phiTy2
),4,4))
```

#DATA generation

```
FFx1<-Factors[,1]; FFy1<-Factors[,2]; Tx<-Factors[,3]; Ty<-Factors[,4];
DT1<-cbind(FFx1,FFy1)+c1*mvrnorm(NN, rep(0,2),matrix(c(psix2,psixy,psixy,psiy2),2,2))+(c(mux1,muy1)+ c2*cbind(Tx,Ty))
FF2<- cbind(betax*FFx1+gammax*FFy1, betay*FFy1+gammay*FFx1)+mvrnorm(NN,rep(0,2), matrix(c(omegax,omegaxy,omegaxy,omegay),2,2));
FFx2<-FF2[,1]; FFy2<-FF2[,2]
DT2<-FF2+c1*mvrnorm(NN, rep(0,2),matrix(c(psix2,psixy,psixy,psiy2),2,2))+(c(mux2,muy2)+ c2*cbind(Tx,Ty))
FF3<- cbind(betax*FFx2+gammax*FFy2, betay*FFy2+gammay*FFx2)+mvrnorm(NN,rep(0,2), matrix(c(omegax,omegaxy,omegaxy,omegay),2,2));
FFx3<-FF3[,1]; FFy3<-FF3[,2]
DT3<-FF3+c1*mvrnorm(NN, rep(0,2),matrix(c(psix2,psixy,psixy,psiy2),2,2))+(c(mux3,muy3)+ c2*cbind(Tx,Ty))
FF4<- cbind(betax*FFx3+gammax*FFy3, betay*FFy3+gammay*FFx3)+mvrnorm(NN,rep(0,2), matrix(c(omegax,omegaxy,omegaxy,omegay),2,2));
FFx4<-FF4[,1]; FFy4<-FF4[,2]
DT4<-FF4+c1*mvrnorm(NN, rep(0,2),matrix(c(psix2,psixy,psixy,psiy2),2,2))+(c(mux4,muy4)+ c2*cbind(Tx,Ty))
FF5<- cbind(betax*FFx4+gammax*FFy4, betay*FFy4+gammay*FFx4)+mvrnorm(NN,rep(0,2), matrix(c(omegax,omegaxy,omegaxy,omegay),2,2));
FFx5<-FF5[,1]; FFy5<-FF5[,2]
DT5<-FF5+c1*mvrnorm(NN, rep(0,2),matrix(c(psix2,psixy,psixy,psiy2),2,2))+(c(mux5,muy5)+ c2*cbind(Tx,Ty))
FF6<- cbind(betax*FFx5+gammax*FFy5, betay*FFy5+gammay*FFx5)+mvrnorm(NN,rep(0,2), matrix(c(omegax,omegaxy,omegaxy,omegay),2,2));
FFx6<-FF6[,1]; FFy6<-FF6[,2]
DT6<-FF6+c1*mvrnorm(NN, rep(0,2),matrix(c(psix2,psixy,psixy,psiy2),2,2))+(c(mux6,muy6)+ c2*cbind(Tx,Ty))
FF7<- cbind(betax*FFx6+gammax*FFy6, betay*FFy6+gammay*FFx6)+mvrnorm(NN,rep(0,2), matrix(c(omegax,omegaxy,omegaxy,omegay),2,2));
FFx7<-FF7[,1]; FFy7<-FF7[,2]
DT7<-FF7+c1*mvrnorm(NN, rep(0,2),matrix(c(psix2,psixy,psixy,psiy2),2,2))+(c(mux7,muy7)+ c2*cbind(Tx,Ty))
FF8<- cbind(betax*FFx7+gammax*FFy7, betay*FFy7+gammay*FFx7)+mvrnorm(NN,rep(0,2), matrix(c(omegax,omegaxy,omegaxy,omegay),2,2));
FFx8<-FF8[,1]; FFy8<-FF8[,2]
DT8<-FF8+c1*mvrnorm(NN, rep(0,2),matrix(c(psix2,psixy,psixy,psiy2),2,2))+(c(mux8,muy8)+ c2*cbind(Tx,Ty))

Data<-data.frame(cbind(DT1,DT2,DT3,DT4,DT5,DT6,DT7,DT8))
colnames(Data)<-c("x1","y1","x2","y2","x3","y3","x4","y4","x5","y5","x6","y6","x7","y7","x8","y8")
DATA<-Data[,1:(2*TT)]
```

#DATA analysis to evaluate Bias, SE and statistical significance.

```
fit<- suppressMessages(suppressWarnings (lavaan(CLPMT4, data = DATA,control=list(iter.max=50)))) #change the red-highlighted part for different T
COU1<-sign(sum(diag(inspect(fit, "coefficients")$psi)<0)+sum(diag(inspect(fit, "coefficients")$theta)<0)+sum(det(fitted(fit)$cov)<0))
CLGSD<- parameterEstimates(fit,standardized=TRUE)[2*TT+9,12];CLG<-inspect(fit, "coefficients")$beta[2+TT,1]
CLAIC<- AIC(fit); CLBIC<-BIC(fit);CLGbias<-inspect(fit, "coefficients")$beta[2+TT,1]-gammax;CLGse<- parameterEstimates(fit,standardized=TRUE)[2*TT+9,6]
SIGCLG<- (sign(0.05-parameterEstimates(fit)[2*TT+9,8])+1)/2

fit<- suppressMessages(suppressWarnings (lavaan(RICLPMT4, data = DATA,control=list(iter.max=50))))#change the red-highlighted part for different T
COU2<-sign(sum(diag(inspect(fit, "coefficients")$psi)<0)+sum(diag(inspect(fit, "coefficients")$theta)<0)+sum(det(fitted(fit)$cov)<0))
RICLGSD<- parameterEstimates(fit,standardized=TRUE)[4*TT+14,12];RICLG<-inspect(fit, "coefficients")$beta[4+TT,3]
RICLAIC<-                AIC(fit);                RICLBIC<-BIC(fit);RICLGbias<-inspect(fit,                "coefficients")$beta[4+TT,3]-gammax;RICLGse<-
parameterEstimates(fit,standardized=TRUE)[4*TT+14,6]
SIGRICLG<- (sign(0.05-parameterEstimates(fit)[4*TT+14,8])+1)/2

fit<- suppressMessages(suppressWarnings(lavaan(STARTST4, data = DATA,control=list(iter.max=50))))#change the red-highlighted part for different T
COU3<-sign(sum(diag(inspect(fit, "coefficients")$psi)<0)+sum(diag(inspect(fit, "coefficients")$theta)<0)+sum(det(fitted(fit)$cov)<0))
STGSD<- parameterEstimates(fit, standardized=TRUE)[7*TT+14,12];STG<-inspect(fit, "coefficients")$beta[4+TT,3]
STAIC<-AIC(fit);STBIC<-BIC(fit);STGbias<-inspect(fit, "coefficients")$beta[4+TT,3]-gammax; STGse<- parameterEstimates(fit,standardized=TRUE)[7*TT+14,6]
SIGSTG<- (sign(0.05-parameterEstimates(fit)[7*TT+14,8])+1)/2

SIGCLRICLtype<-2*SIGCLG+(SIGRICLG+1)
SIGCLSTtype<-2*SIGCLG+(SIGSTG+1)
SIGRICLSTtype<-2*SIGRICLG+(SIGSTG+1)

if((1-COU1)*(1-COU2)*(1-COU3)>0){
AIC1ES<-AIC1ES+ifelse(rank(c(CLAIC,RICLAIC,STAIC))[1]==1,1,0)
BIC1ES<- BIC1ES+ifelse(rank(c(CLBIC,RICLBIC,STBIC))[1]==1,1,0)
AIC2ES<-AIC2ES+ifelse(rank(c(CLAIC,RICLAIC,STAIC))[2]==1,1,0)
BIC2ES<- BIC2ES+ifelse(rank(c(CLBIC,RICLBIC,STBIC))[2]==1,1,0)
AIC3ES<-AIC3ES+ifelse(rank(c(CLAIC,RICLAIC,STAIC))[3]==1,1,0)
BIC3ES<- BIC3ES+ifelse(rank(c(CLBIC,RICLBIC,STBIC))[3]==1,1,0)
ttt<-ttt+1
CLGSDES[ttt]<-CLGSD
CLGES[ttt]<-CLG
RICLGSDES[ttt]<-RICLGSD
RICLGES[ttt]<-RICLG
STGSDES[ttt]<-STGSD
STGES[ttt]<-STG
CLGESbias[ttt]<-CLGbias
CLGESse[ttt]<-CLGse
RICLGESbias[ttt]<-RICLGbias
RICLGESse[ttt]<-RICLGse
STGESbias[ttt]<-STGbias
STGESse[ttt]<-STGse
sumSIGDiffCLRICLG1<- sumSIGDiffCLRICLG1+ifelse(SIGCLRICLtype==1,1,0)
sumSIGDiffCLRICLG2<- sumSIGDiffCLRICLG2+ifelse(SIGCLRICLtype==2,1,0)
sumSIGDiffCLRICLG3<- sumSIGDiffCLRICLG3+ifelse(SIGCLRICLtype==3,1,0)
sumSIGDiffCLRICLG4<- sumSIGDiffCLRICLG4+ifelse(SIGCLRICLtype==4,1,0)
sumSIGDiffCLSTG1<- sumSIGDiffCLSTG1+ifelse(SIGCLSTtype==1,1,0)
sumSIGDiffCLSTG2<- sumSIGDiffCLSTG2+ifelse(SIGCLSTtype==2,1,0)
sumSIGDiffCLSTG3<- sumSIGDiffCLSTG3+ifelse(SIGCLSTtype==3,1,0)
sumSIGDiffCLSTG4<- sumSIGDiffCLSTG4+ifelse(SIGCLSTtype==4,1,0)
sumSIGDiffRICLSTG1<- sumSIGDiffRICLSTG1+ifelse(SIGRICLSTtype==1,1,0)
sumSIGDiffRICLSTG2<- sumSIGDiffRICLSTG2+ifelse(SIGRICLSTtype==2,1,0)
sumSIGDiffRICLSTG3<- sumSIGDiffRICLSTG3+ifelse(SIGRICLSTtype==3,1,0)
sumSIGDiffRICLSTG4<- sumSIGDiffRICLSTG4+ifelse(SIGRICLSTtype==4,1,0)
}else{
}

}

  RESULT[3*2*3*(aaaa-1)+2*3*(cccc-1)+3*(bbbb-1)+dddd,1]<-mean(CLGSDES)
  RESULT[3*2*3*(aaaa-1)+2*3*(cccc-1)+3*(bbbb-1)+dddd,2]<-mean(RICLGSDES)
  RESULT[3*2*3*(aaaa-1)+2*3*(cccc-1)+3*(bbbb-1)+dddd,3]<-mean(STGSDES)
  RESULT[3*2*3*(aaaa-1)+2*3*(cccc-1)+3*(bbbb-1)+dddd,4]<-mean(CLGES)
  RESULT[3*2*3*(aaaa-1)+2*3*(cccc-1)+3*(bbbb-1)+dddd,5]<-mean(RICLGES)
  RESULT[3*2*3*(aaaa-1)+2*3*(cccc-1)+3*(bbbb-1)+dddd,6]<-mean(STGES)
  RESULT[3*2*3*(aaaa-1)+2*3*(cccc-1)+3*(bbbb-1)+dddd,7]<-mean(CLGESse)
  RESULT[3*2*3*(aaaa-1)+2*3*(cccc-1)+3*(bbbb-1)+dddd,8]<-mean(RICLGESse)
  RESULT[3*2*3*(aaaa-1)+2*3*(cccc-1)+3*(bbbb-1)+dddd,9]<-mean(STGESse)
RESULT[3*2*3*(aaaa-1)+2*3*(cccc-1)+3*(bbbb-1)+dddd,10] <-mean(CLGESbias)
  RESULT[3*2*3*(aaaa-1)+2*3*(cccc-1)+3*(bbbb-1)+dddd,11] <-mean(RICLGESbias)
  RESULT[3*2*3*(aaaa-1)+2*3*(cccc-1)+3*(bbbb-1)+dddd,12] <- mean(STGESbias)
RESULT[3*2*3*(aaaa-1)+2*3*(cccc-1)+3*(bbbb-1)+dddd,13] <-AIC1ES
RESULT[3*2*3*(aaaa-1)+2*3*(cccc-1)+3*(bbbb-1)+dddd,14] <-AIC2ES
RESULT[3*2*3*(aaaa-1)+2*3*(cccc-1)+3*(bbbb-1)+dddd,15] <-AIC3ES
RESULT[3*2*3*(aaaa-1)+2*3*(cccc-1)+3*(bbbb-1)+dddd,16] <-BIC1ES
RESULT[3*2*3*(aaaa-1)+2*3*(cccc-1)+3*(bbbb-1)+dddd,17] <-BIC2ES
RESULT[3*2*3*(aaaa-1)+2*3*(cccc-1)+3*(bbbb-1)+dddd,18] <-BIC3ES
RESULT[3*2*3*(aaaa-1)+2*3*(cccc-1)+3*(bbbb-1)+dddd,19] <- sd(CLGESbias)
  RESULT[3*2*3*(aaaa-1)+2*3*(cccc-1)+3*(bbbb-1)+dddd,20] <- sd(RICLGESbias)
  RESULT[3*2*3*(aaaa-1)+2*3*(cccc-1)+3*(bbbb-1)+dddd,21] <- sd(STGESbias)
  RESULT[3*2*3*(aaaa-1)+2*3*(cccc-1)+3*(bbbb-1)+dddd,22]<-mean(CLGES-RICLGES)
  RESULT[3*2*3*(aaaa-1)+2*3*(cccc-1)+3*(bbbb-1)+dddd,23] <-mean(CLGES-STGES)
  RESULT[3*2*3*(aaaa-1)+2*3*(cccc-1)+3*(bbbb-1)+dddd,24] <-mean(RICLGES-STGES)
  RESULT[3*2*3*(aaaa-1)+2*3*(cccc-1)+3*(bbbb-1)+dddd,25]<-sd(CLGES-RICLGES)
  RESULT[3*2*3*(aaaa-1)+2*3*(cccc-1)+3*(bbbb-1)+dddd,26] <-sd(CLGES-STGES)
  RESULT[3*2*3*(aaaa-1)+2*3*(cccc-1)+3*(bbbb-1)+dddd,27] <-sd(RICLGES-STGES)
  RESULT[3*2*3*(aaaa-1)+2*3*(cccc-1)+3*(bbbb-1)+dddd,28]<-sumSIGDiffCLRICLG1/Repeat
  RESULT[3*2*3*(aaaa-1)+2*3*(cccc-1)+3*(bbbb-1)+dddd,29]<-sumSIGDiffCLRICLG2/Repeat
  RESULT[3*2*3*(aaaa-1)+2*3*(cccc-1)+3*(bbbb-1)+dddd,30]<-sumSIGDiffCLRICLG3/Repeat
  RESULT[3*2*3*(aaaa-1)+2*3*(cccc-1)+3*(bbbb-1)+dddd,31]<-sumSIGDiffCLRICLG4/Repeat
  RESULT[3*2*3*(aaaa-1)+2*3*(cccc-1)+3*(bbbb-1)+dddd,32]<-sumSIGDiffCLSTG1/Repeat
  RESULT[3*2*3*(aaaa-1)+2*3*(cccc-1)+3*(bbbb-1)+dddd,33]<-sumSIGDiffCLSTG2/Repeat
  RESULT[3*2*3*(aaaa-1)+2*3*(cccc-1)+3*(bbbb-1)+dddd,34]<-sumSIGDiffCLSTG3/Repeat
  RESULT[3*2*3*(aaaa-1)+2*3*(cccc-1)+3*(bbbb-1)+dddd,35]<-sumSIGDiffCLSTG4/Repeat
  RESULT[3*2*3*(aaaa-1)+2*3*(cccc-1)+3*(bbbb-1)+dddd,36]<-sumSIGDiffRICLSTG1/Repeat
  RESULT[3*2*3*(aaaa-1)+2*3*(cccc-1)+3*(bbbb-1)+dddd,37]<-sumSIGDiffRICLSTG2/Repeat
  RESULT[3*2*3*(aaaa-1)+2*3*(cccc-1)+3*(bbbb-1)+dddd,38]<-sumSIGDiffRICLSTG3/Repeat
  RESULT[3*2*3*(aaaa-1)+2*3*(cccc-1)+3*(bbbb-1)+dddd,39]<-sumSIGDiffRICLSTG4/Repeat
RESULT[3*2*3*(aaaa-1)+2*3*(cccc-1)+3*(bbbb-1)+dddd,40]<-gammax
RESULT[3*2*3*(aaaa-1)+2*3*(cccc-1)+3*(bbbb-1)+dddd,41]<-betax
RESULT[3*2*3*(aaaa-1)+2*3*(cccc-1)+3*(bbbb-1)+dddd,42]<-psix2
RESULT[3*2*3*(aaaa-1)+2*3*(cccc-1)+3*(bbbb-1)+dddd,43]<-NN
};};};}
write.csv(RESULT,"Specifying an appropriate directory and file name here")
}
```

Table A Full references for retained papers.

ID	References
1	Adachi, P. J., & Willoughby, T. (2016). The longitudinal association between competitive video game play and aggression among adolescents and young adults. *Child development*, *87*, 1877-1892.
2	Andrade, F. H. (2014). Co-occurrences between adolescent substance use and academic performance: School context influences a multilevel-longitudinal perspective. *Journal of Adolescence*, *37*, 953-963.
3	Arnett, A. B., Pennington, B. F., Willcutt, E., Dmitrieva, J., Byrne, B., Samuelsson, S., & Olson, R. K. (2012). A cross-lagged model of the development of ADHD inattention symptoms and rapid naming speed. *Journal of Abnormal Child Psychology*, *40*, 1313-1326.
4	Arnett, A. B., Pennington, B. F., Young, J. F., & Hankin, B. L (2016). Links between within-person fluctuations in hyperactivity/attention problems and subsequent conduct problems. *Journal of Child Psychology and Psychiatry*, *57*, 502-509.
5	Ayalon, L., Shiovitz-Ezra, S., & Roziner, I. (2016). A cross-lagged model of the reciprocal associations of loneliness and memory functioning. *Psychology and Aging*, *31*, 255-261.
6	Baams, L., Overbeek, G., Dubas, J. S., Doornwaard, S. M., Rommes, E., & Van Aken, M. A. (2015). Perceived realism moderates the relation between sexualized media consumption and permissive sexual attitudes in Dutch adolescents. *Archives of Sexual Behavior*, *44*, 743-754.
7	Besemer, S., Loeber, R., Hinshaw, S. P., & Pardini, D. A. (2016). Bidirectional Associations Between Externalizing Behavior Problems and Maladaptive Parenting Within Parent-Son Dyads Across Childhood. *Journal of Abnormal Child Psychology*, *44*, 1387-1398.
8	Banerjee, R., Watling, D., & Caputi, M. (2011). Peer relations and the understanding of faux pas: Longitudinal evidence for bidirectional associations. *Child Development*, *82*, 1887-1905.
9	Baydar, N., & Akcinar, B. (2018). Reciprocal relations between the trajectories of mothers' harsh discipline, responsiveness and aggression in early childhood. *Journal of Abnormal Child Psychology*, *46*, 83-97.
10	Beaujean, A. A., Parker, S., & Qiu, X. (2013). The relationship between cognitive ability and depression: a longitudinal data analysis. *Social Psychiatry and Psychiatric Epidemiology*, *48*, 1983-1992.
11	Bekkhus, M., Rutter, M., Barker, E. D., & Borge, A. I. (2011). The role of pre-and postnatal timing of family risk factors on child behavior at 36 months. *Journal of Abnormal Child Psychology*, *39*, 611-621.

12 Bennett, T. A., Szatmari, P., Georgiades, K., Hanna, S., Janus, M., Georgiades, ... & Mirenda, P. (2015). Do reciprocal associations exist between social and language pathways in preschoolers with autism spectrum disorders?. *Journal of Child Psychology and Psychiatry*, *56*, 874-883.

13 Bentley, J. P., Brown, C. J., McGwin, G., Sawyer, P., Allman, R. M., & Roth, D. L. (2013). Functional status, life-space mobility, and quality of life: a longitudinal mediation analysis. *Quality of Life Research*, *22*, 1621-1632.

14 Best, J. R., Davis, J. C., & Liu-Ambrose, T. (2015). Longitudinal analysis of physical performance, functional status, physical activity, and mood in relation to executive function in older adults who fall. *Journal of the American Geriatrics Society*, *63*, 1112-1120.

15 Birkeland, M. S., Nielsen, M. B., Knardahl, S., & Heir, T. (2016). Time-lagged relationships between leadership behaviors and psychological distress after a workplace terrorist attack. *International Archives of Occupational and Environmental Health*, *89*, 689-697.

16 Bohlmann, N. L., Maier, M. F., & Palacios, N. (2015). Bidirectionality in self-regulation and expressive vocabulary: Comparisons between monolingual and dual language learners in preschool. *Child Development*, *86*, 1094-1111.

17 Bolhuis, K., McAdams, T. A., Monzani, B., Gregory, A. M., Mataix-Cols, D., Stringaris, A., & Eley, T. C. (2014). Aetiological overlap between obsessive–compulsive and depressive symptoms: a longitudinal twin study in adolescents and adults. *Psychological Medicine*, *44*, 1439-1449.

18 Bolhuis, K., Lubke, G. H., van der Ende, J., Bartels, M., van Beijsterveldt, C. E., Lichtenstein, P., ... & Boomsma, D. I. (2017). Disentangling heterogeneity of childhood disruptive behavior problems into dimensions and subgroups. *Journal of the American Academy of Child & Adolescent Psychiatry*, *56*, 678-686.

19 Bondü, R., Rothmund, T., & Gollwitzer, M. (2016). Mutual long-term effects of school bullying, victimization, and justice sensitivity in adolescents. *Journal of Adolescence*, *48*, 67-72.

20 Bonvanie, I. J., Oldehinkel, A. J., Rosmalen, J. G., & Janssens, K. A. (2016). Sleep problems and pain: a longitudinal cohort study in emerging adults. *Pain*, *157*, 957-963.

21 Bourque, J., O'Leary-Barrett, M., & Conrod, P. (2016). 6.88 THE IMPACT OF CANNABIS USE AND EMERGING PSYCHOTIC EXPERIENCES EXPLAINED BY SLEEP PROBLEMS AND ANXIETY SYMPTOMS. *Journal of the American Academy of Child & Adolescent Psychiatry*, *55*, S233.

22 Boyes, M. E., Bowes, L., Cluver, L. D., Ward, C. L., & Badcock, N. A. (2014). Bullying victimisation, internalising symptoms, and conduct problems in South African children and adolescents: A longitudinal investigation. *Journal of Abnormal Child Psychology*, *42*, 1313-1324.

23 Boylan, K., Georgiades, K., & Szatmari, P. (2010). The longitudinal association between oppositional and depressive symptoms across childhood. *Journal of the American Academy of Child & Adolescent Psychiatry*, *49*, 152-161.

24 Breeman, L. D., Van Lier, P. A. C., Wubbels, T., Verhulst, F. C., van der Ende, J., Maras, A., Hopman, J. A. B., & Tick, N. T. (2015). Developmental links between disobedient behavior and social classroom relationships in boys with psychiatric disorders in special education. *Journal of Abnormal Child Psychology*, *43*, 787-799.

25 Brière, F. N., Rohde, P., Seeley, J. R., Klein, D., & Lewinsohn, P. M. (2014). Comorbidity between major depression and alcohol use disorder from adolescence to adulthood. *Comprehensive Psychiatry*, *55*, 526-533.

26 Te Brinke, L. W., Deković, M., Stoltz, S. E., & Cillessen, A. H. (2017). Bidirectional effects between parenting and aggressive child behavior in the context of a preventive intervention. *Journal of Abnormal Child Psychology*, *45*, 921-934.

27 Brown, S. C., Huang, S., Perrino, T., Surio, P., Borges-Garcia, R., Flavin, K., Brown, C. H., Pantin, H., & Szapocznik, J. (2011). The relationship of perceived neighborhood social climate to walking in Hispanic older adults: a longitudinal, cross-lagged panel analysis. *Journal of Aging and Health*, *23*, 1325-1351.

28 Gerhart, J. I., Burns, J. W., Post, K. M., Smith, D. A., Porter, L. S., Burgess, H. J., ... & Keefe, F. J. (2016). Relationships between sleep quality and pain-related factors for people with chronic low back pain: tests of reciprocal and time of day effects. *Annals of Behavioral Medicine*, *51*, 365-375.

29 Calvete, E., Gamez-Guadix, M., & Garcia-Salvador, S. (2015). Social information processing in child-to-parent aggression: Bidirectional associations in a 1-year prospective study. *Journal of Child and Family Studies*, *24*, 2204-2216.

30 Calvete, E., Orue, I., & Gámez-Guadix, M. (2015). Reciprocal longitudinal associations between substance use and child-to-parent violence in adolescents. *Journal of Adolescence*, *44*, 124-133.

31 Chang, H., & Shaw, D. S. (2016). The emergence of parent–child coercive processes in toddlerhood. *Child Psychiatry & Human Development*, *47*, 226-235.

32 Chen, X., Huang, X., Wang, L., & Chang, L. (2012). Aggression, peer relationships, and depression in Chinese children: a multiwave longitudinal study. *Journal of Child Psychology and Psychiatry*, *53*, 1233-1241.

33 Chen, J., Zhou, X., Zeng, M., & Wu, X. (2015). Post-traumatic stress symptoms and post-traumatic growth: Evidence from a longitudinal study following an earthquake disaster. *PLoS ONE*, *10*, e0127241.

34 Cheng, J., East, P., Blanco, E., Kang Sim, E., Castillo, M., Lozoff, B., & Gahagan, S. (2016). Obesity leads to declines in motor skills across childhood. *Child: care, health and development*, *42*, 343-350.

35 Chi, P., Li, X., Zhao, J., & Zhao, G. (2014). Vicious circle of perceived stigma, enacted stigma and depressive symptoms among children affected by HIV/AIDS in China. *AIDS and Behavior*, *18*, 1054-1062.

36 Choi, K., Forster, J., Erickson, D., Lazovich, D., & Southwell, B. G. (2012). The reciprocal relationships between changes in adolescent perceived prevalence of smoking in movies and progression of smoking status. *Tobacco Control*, *21*, 492-496.

37 Christensen, J. O., & Knardahl, S. (2012). Work and headache: a prospective study of psychological, social, and mechanical predictors of headache severity. *Pain*, *153*, 2119-2132.

38 Conway, A., Miller, A. L., & Modrek, A. (2017). Testing reciprocal links between trouble getting to sleep and internalizing behavior problems, and bedtime resistance and externalizing behavior problems in toddlers. *Child Psychiatry & Human Development*, *48*, 678-689.

39 Cooley, J. L., Fite, P. J., & Pederson, C. A. (2018). Bidirectional associations between peer victimization and functions of aggression in middle childhood: further evaluation across informants and academic years. *Journal of Abnormal Child Psychology*, *46*, 99-111.

40 Cowlishaw, S., Niele, S., Teshuva, K., Browning, C., & Kendig, H. (2013). Older adults' spirituality and life satisfaction: a longitudinal test of social support and sense of coherence as mediating mechanisms. *Ageing & Society*, *33*, 1243-1262.

41 Crocetti, E., Moscatelli, S., Van der Graaff, J., Keijsers, L., Van Lier, P., Koot, H. M., Rubini, M., Meeus, W., & Branje, S. (2016). The dynamic interplay among maternal empathy, quality of mother-adolescent relationship, and adolescent antisocial behaviors: new insights from a six-wave longitudinal multi-informant study. *PLoS ONE*, *11*, e0150009.

42 Crocetti, E., Branje, S., Rubini, M., Koot, H. M., & Meeus, W. (2017). Identity processes and parent–child and sibling relationships in adolescence: A five-wave multi-informant longitudinal study. *Child Development*, *88*, 210-228.

43 Crosnoe, R., Benner, A. D., & Schneider, B. (2012). Drinking, socioemotional functioning, and academic progress in secondary school. *Journal of Health and Social Behavior*, *53*, 150-164.

44 Dakanalis, A., Carrà, G., Calogero, R., Fida, R., Clerici, M., Zanetti, M. A., & Riva, G. (2015). The developmental effects of media-ideal internalization and self-objectification processes on adolescents' negative body-feelings, dietary restraint, and binge eating. *European Child & Adolescent Psychiatry*, *24*, 997-1010.

45 Dakanalis, A., Clerici, M., & Carrà, G. (2016). Narcissistic vulnerability and grandiosity as mediators between insecure attachment and future eating disordered behaviors: a prospective analysis of over 2,000 freshmen. *Journal of Clinical Psychology*, *72*, 279-292.

46 Daniel, E., Dys, S. P., Buchmann, M., & Malti, T. (2014). Developmental relations between sympathy, moral emotion attributions, moral reasoning, and social justice values from childhood to early adolescence. *Journal of Adolescence*, *37*, 1201-1214.

47 Daniel, E., Plamondon, A., & Jenkins, J. M. (2018). An examination of the sibling training hypothesis for disruptive behavior in early childhood. *Child Development*, *89*, 235-247.

48 Danzo, S., Connell, A. M., & Stormshak, E. A. (2017). Associations between alcohol-use and depression symptoms in adolescence: Examining gender differences and pathways over time. *Journal of Adolescence*, *56*, 64-74.

49 Das, A., & Sawin, N. (2016). Social modulation or hormonal causation? Linkages of testosterone with sexual activity and relationship quality in a nationally representative longitudinal sample of older adults. *Archives of Sexual Behavior*, *45*, 2101-2115.

50 De Laet, S., Doumen, S., Vervoort, E., Colpin, H., Van Leeuwen, K., Goossens, L., & Verschueren, K. (2014). Transactional links between teacher–child relationship quality and perceived versus sociometric popularity: A three-wave longitudinal study. *Child Development*, *85*, 1647-1662.

51 de Leeuw, R. N., Sargent, J. D., Stoolmiller, M., Scholte, R. H., Engels, R. C., & Tanski, S. E. (2011). Association of smoking onset with R-rated movie restrictions and adolescent sensation seeking. *Pediatrics*, *127*, e96-e105.

52 de Wilde, A., Koot, H. M., & van Lier, P. A. (2016). Developmental links between children's working memory and their social relations with teachers and peers in the early school years. *Journal of Abnormal Child Psychology*, *44*, 19-30.

53 Dempsy, J., McQuillin, S., Bulter, A. M., & Axelrad, M. E. (2016). Maternal depression and parent management training outcomes. *Journal of Clinical Psychology in Medical Settings*, *23*, 240-246.

54 Deschênes, S. S., Burns, R. J., & Schmitz, N. (2016). Anxiety symptoms and functioning in a commyunity sample of individuals weth type 2 diabetes: A longitudinal study. *Journal of Diabetes*, *8*, 854-862.

55 Diamantopoulou, S., Verhulst, F. C., & Van der Ende, J. (2011). The parallel development of ODD and CD symptoms from early childhood to adolescence. *European Child & Adolescent Psychiatry*, *20*, 301-309.

56 Ding, X., Tang, R., Jiang, C., & Tang, Y. Y. (2014). Modeling emotion-creativity interaction following brief training. *BMC Neuroscience*, *15*, P32.

57 Ding, X., Tang, Y. Y., Tang, R., & Posner, M. I. (2014). Improving creativity performance by short-term meditation. *Behavioral and Brain Functions*, *10*, 9.

58 Doane, M. J., & Elliott, M. (2016). Religiosity and self-rated health: A longitudinal examination of their reciprocal effects. *Journal of Religion and Health*, *55*, 844-855.

59 Eggers, S. M., Mathews, C., Aarø, L. E., McClinton-Appollis, T., Bos, A. E., & de Vries, H. (2017). Predicting primary and secondary abstinence among adolescent boys and girls in the Western Cape, South Africa. *AIDS and Behavior*, *21*, 1417-1428.

60 Fabbri, E., An, Y., Schrack, J. A., Gonzalez-Freire, M., Zoli, M., Simonsick, E. M., Guralnik, J. M., Boyd, C. M., Studenski, S. A., & Ferrucci, L. (2015). Energy metabolism and the burden of multimorbidity in older adults: results of the Baltimore longitudinal study of aging. *Journals of Gerontology Series A: Biological Sciences and Medical Sciences*, *70*, 1297-1303.

61 Faller, H., Strahl, A., Richard, M., Niehues, C., & Meng, K. (2017). The prospective relationship between satisfaction with information and symptoms of depression and anxiety in breast cancer: A structural equation modeling analysis. *Psycho-oncology*, *26*, 1741-1748.

62 Fanti, K. A., & Centifanti, L. C. M. (2014). Childhood callous-unemotional traits moderate the relation between parenting distress and conduct problems over time. *Child Psychiatry & Human Development*, *45*, 173-184.

63 Ferreiro, F., Wichstrøm, L., Seoane, G., & Senra, C. (2014). Reciprocal associations between depressive symptoms and disordered eating among adolescent girls and boys: A multiwave, prospective study. *Journal of Abnormal Child Psychology*, *42*, 803-812.

64 Feldt, T., Hyvönen, K., Mäkikangas, A., Rantanen, J., Huhtala, M., & Kinnunen, U. (2016). Overcommitment as a predictor of effort—reward imbalance: evidence from an 8-year follow-up study. *Scandinavian Journal of Work, Environment & Health*, *42*, 309-319.

65 Fielder, R. L., Walsh, J. L., Carey, K. B., & Carey, M. P. (2014). Sexual hookups and adverse health outcomes: A longitudinal study of first-year college women. *Journal of Sex Research*, *51*, 131-144.

66 Fletcher, A. C., & Johnston, C. A. (2016). Parenting behaviors and child externalizing: A short-term investigation of directionality. *Journal of Child and Family Studies*, *25*, 3150-3159.

67 Flouri, E., Narayanan, M. K., & Midouhas, E. (2015). The cross-lagged relationship between father absence and child problem behaviour in the early years. *Child: care, health and development*, *41*, 1090-1097.

68 Flouri, E., Midouhas, E., & Narayanan, M. K. (2016). The relationship between father involvement and child problem behaviour in intact families: A 7-year cross-lagged study. *Journal of Abnormal Child Psychology*, *44*, 1011-1021.

69 Flournoy, J. C., Pfeifer, J. H., Moore, W. E., Tackman, A. M., Masten, C. L., Mazziotta, J. C., Iacoboni, M., & Dapretto, M. (2016). Neural reactivity to emotional faces may mediate the relationship between childhood empathy and adolescent prosocial behavior. *Child Development*, *87*, 1691-1702.

70 Foti, D. J., Kotov, R., Guey, L. T., & Bromet, E. J. (2010). Cannabis use and the course of schizophrenia: 10-year follow-up after first hospitalization. *American Journal of Psychiatry*, *167*, 987-993.

71 Freedman, S. A., Gilad, M., Ankri, Y., Roziner, I., & Shalev, A. Y. (2015). Social relationship satisfaction and PTSD: which is the chicken and which is the egg?. *European Journal of Psychotraumatology*, *6*, 28864.

72 French, D. C., Christ, S., Lu, T., & Purwono, U. (2014). Trajectories of Indonesian adolescents' religiosity, problem behavior, and friends' religiosity: Covariation and sequences. *Child Development*, *85*, 1643-1646.

73 Frijns, T., Keijsers, L., Branje, S., & Meeus, W. (2010). What parents don't know and how it may affect their children: Qualifying the disclosure–adjustment link. *Journal of Adolescence*, *33*, 261-270.

74 Fuller-Tyszkiewicz, M., McCabe, M., Skouteris, H., Richardson, B., Nihill, K., Watson, B., & Solomon, D. (2015). Does body satisfaction influence self-esteem in adolescents' daily lives? An experience sampling study. *Journal of Adolescence*, *45*, 11-19.

75 Garbarski, D. (2014). The interplay between child and maternal health: reciprocal relationships and cumulative disadvantage during childhood and adolescence. *Journal of Health and Social Behavior*, *55*, 91-106.

76 Garon‐Carrier, G., Boivin, M., Guay, F., Kovas, Y., Dionne, G., Lemelin, J. P., Séguin, J. R., Vitaro, F., & Tremblay, R. E. (2016). Intrinsic motivation and achievement in mathematics in elementary school: A longitudinal investigation of their association. *Child Development*, *87*, 165-175.

77 Gershoff, E. T., Lansford, J. E., Sexton, H. R., Davis‐Kean, P., & Sameroff, A. J. (2012). Longitudinal links between spanking and children’s externalizing behaviors in a national sample of White, Black, Hispanic, and Asian American families. *Child Development*, *83*, 838-843.

78 Girard, L. C., Pingault, J. B., Doyle, O., Falissard, B., & Tremblay, R. E. (2016). Developmental Associations Between Conduct Problems and Expressive Language in Early Childhood: A Population-Based Study. *Journal of Abnormal Child Psychology*, *44*, 1033-1043.

79 Girard, L. C., Doyle, O., & Tremblay, R. E. (2017). Maternal warmth and toddler development: support for transactional models in disadvantaged families. *European Child & Adolescent Psychiatry*, *26*, 497-507.

80 Girard, L. C., Pingault, J. B., Falissard, B., Boivin, M., Dionne, G., & Tremblay, R. E. (2014). Physical aggression and language ability from 17 to 72 months: Cross-lagged effects in a population sample. *PLoS ONE*, *9*, e112185.

81 Good, M., Hamza, C., & Willoughby, T. (2017). A longitudinal investigation of the relation between nonsuicidal self-injury and spirituality/religiosity. *Psychiatry Research*, *250*, 106-112.

82 Goodman, S. H., Lusby, C. M., Thompson, K., Newport, D. J., & Stowe, Z. N. (2014). MATERNAL DEPRESSION IN ASSOCIATION WITH FATHERS’INVOLVEMENT WITH THEIR INFANTS: SPILLOVER OR COMPENSATION/BUFFERING?. *Infant Mental Health Journal*, *35*, 495-508.

83 Greven, C. U., Rijsdijk, F. V., Asherson, P., & Plomin, R. (2012). A longitudinal twin study on the association between ADHD symptoms and reading. *Journal of Child Psychology and Psychiatry*, *53*, 234-242.

84 Greven, C. U., Asherson, P., Rijsdijk, F. V., & Plomin, R. (2011). A longitudinal twin study on the association between inattentive and hyperactive-impulsive ADHD symptoms. *Journal of Abnormal Child Psychology*, *39*, 623-632.

85 Gudmundsson, P., Lindwall, M., Gustafson, D. R., Östling, S., Hällström, T., Waern, M., & Skoog, I. (2015). Longitudinal associations between physical activity and depression scores in Swedish women followed 32 years. *Acta Psychiatrica Scandinavica*, *132*, 451-458.

86 Gutenbrunner, C., Salmon, K., & Jose, P. E. (2018). Do overgeneral autobiographical memories predict increased psychopathological symptoms in community youth? A 3-year longitudinal investigation. *Journal of Abnormal Child Psychology*, *46*, 197-208.

87 Hale III, W. W., Keijsers, L., Klimstra, T. A., Raaijmakers, Q. A., Hawk, S., Branje, S. J., Frijns, T., Wijsbroek, S. A. M., van Lier, P., & Meeus, W. H. (2011). How does longitudinally measured maternal expressed emotion affect internalizing and externalizing symptoms of adolescents from the general community?. *Journal of Child Psychology and Psychiatry*, *52*, 1174-1183.

88 Hale III, W. W., Crocetti, E., Nelemans, S. A., Branje, S. J., van Lier, P. A., Koot, H. M., & Meeus, W. H. (2016). Mother and adolescent expressed emotion and adolescent internalizing and externalizing symptom development: a six-year longitudinal study. *European Child & Adolescent Psychiatry*, *25*, 615-624.

89 Hall, B. J., Saltzman, L. Y., Canetti, D., & Hobfoll, S. E. (2015). A longitudinal investigation of the relationship between posttraumatic stress symptoms and posttraumatic growth in a cohort of Israeli Jews and Palestinians during ongoing violence. *PLoS ONE*, *10*, e0124782.

90 Hallett, V., Ronald, A., Rijsdijk, F., & Happé, F. (2010). Association of autistic-like and internalizing traits during childhood: a longitudinal twin study. *American Journal of Psychiatry*, *167*, 809-817.

91 Hamama-Raz, Y., Shrira, A., Ben-Ezra, M., & Palgi, Y. (2015). The recursive effects of quality of life and functional limitation among older adult cancer patients: evidence from the Survey of Health, Ageing and Retirement in Europe. *European Journal of Cancer Care*, *24*, 205-212.

92 Hannigan, L. J., McAdams, T. A., & Eley, T. C. (2017). Developmental change in the association between adolescent depressive symptoms and the home environment: results from a longitudinal, genetically informative investigation. *Journal of Child Psychology and Psychiatry*, *58*, 787-797.

93 Hanson, L. L. M., Peristera, P., Chungkham, H. S., & Westerlund, H. (2016). Longitudinal mediation modeling of unhealthy behaviors as mediators between workplace demands/support and depressive symptoms. *PLoS ONE*, *11*, e0169276.

94 Magnusson, H. L. L., Madsen, I. E., Rugulies, R., Peristera, P., Westerlund, H., & Descatha, A. (2017). Temporal relationships between job strain and low-back pain. *Scandinavian Journal of Work, Environment & Health*, *43*, 396-404.

95 Harlaar, N., Deater-Deckard, K., Thompson, L. A., DeThorne, L. S., & Petrill, S. A. (2011). Associations between reading achievement and independent reading in early elementary school: A genetically informative cross-lagged study. *Child Development*, *82*, 2123-2137.

96 Harris, M. A., Gruenenfelder-Steiger, A. E., Ferrer, E., Donnellan, M. B., Allemand, M., Fend, H., Conger, R. D., & Trzesniewski, K. H. (2015). Do parents foster self-esteem? Testing the prospective impact of parent closeness on adolescent self-esteem. *Child Development*, *86*, 995-1013.

97 Harvey, E. A., Breaux, R. P., & Lugo-Candelas, C. I. (2016). Early development of comorbidity between symptoms of attention-deficit/hyperactivity disorder (ADHD) and oppositional defiant disorder (ODD). *Journal of Abnormal Psychology*, *125*, 154-167.

98 Henchoz, Y., Baggio, S., N'Goran, A. A., Studer, J., Deline, S., Mohler-Kuo, M., Daeppen, J-B., & Gmel, G. (2014). Health impact of sport and exercise in emerging adult men: a prospective study. *Quality of Life Research*, *23*, 2225-2234.

99 Hiemstra, M., Engels, R. C., Barker, E. D., van Schayck, O. C., & Otten, R. (2013). Smoking-specific parenting and smoking onset in adolescence: the role of genes from the dopaminergic system (DRD2, DRD4, DAT1 genotypes). *PLoS ONE*, *8*, e61673.

100 Hietanen, H., Aartsen, M., Kiuru, N., Lyyra, T. M., & Read, S. (2016). Social engagement from childhood to middle age and the effect of childhood socio-economic status on middle age social engagement: results from the National Child Development study. *Ageing & Society*, *36*, 482-507.

101 Hill, P. L., Allemand, M., Grob, S. Z., Peng, A., Morgenthaler, C., & Käppler, C. (2013). Longitudinal relations between personality traits and aspects of identity formation during adolescence. *Journal of Adolescence*, *36*, 413-421.

102 Hinnant, J. B., El-Sheikh, M., Keiley, M., & Buckhalt, J. A. (2013). Marital conflict, allostatic load, and the development of children's fluid cognitive performance. *Child Development*, *84*, 2003-2014.

103 Hipwell, A. E., Stepp, S., Feng, X., Burke, J., Battista, D. R., Loeber, R., & Keenan, K. (2011). Impact of oppositional defiant disorder dimensions on the temporal ordering of conduct problems and depression across childhood and adolescence in girls. *Journal of Child Psychology and Psychiatry*, *52*, 1099-1108.

104 Holmes, C. J., Kim-Spoon, J., & Deater-Deckard, K. (2016). Linking executive function and peer problems from early childhood through middle adolescence. *Journal of Abnormal Child Psychology*, *44*, 31-42.

105 Hopwood, C. J., Donnellan, M. B., & Zanarini, M. C. (2010). Temperamental and acute symptoms of borderline personality disorder: associations with normal personality traits and dynamic relations over time. *Psychological Medicine*, *40*, 1871-1878.

106 Houkes, I., Winants, Y., Twellaar, M., & Verdonk, P. (2011). Development of burnout over time and the causal order of the three dimensions of burnout among male and female GPs. A three-wave panel study. *BMC Public Health*, *11*, 240.

107 Howarth, G. Z., Fettig, N. B., Curby, T. W., & Bell, M. A. (2016). Frontal electroencephalogram asymmetry and temperament across infancy and early childhood: An exploration of stability and bidirectional relations. *Child development*, *87*, 465-476.

108 Huizink, A. C., Menting, B., Oosterman, M., Verhage, M. L., Kunseler, F. C., & Schuengel, C. (2014). The interrelationship between pregnancy-specific anxiety and general anxiety across pregnancy: a longitudinal study. *Journal of Psychosomatic Obstetrics & Gynecology*, *35*, 92-100.

109 Husby, S. M., & Wichstrøm, L. (2017). Interrelationships and continuities in symptoms of oppositional defiant and conduct disorders from age 4 to 10 in the community. *Journal of Abnormal Child Psychology*, *45*, 947-958.

110 Huyghebaert, T., Fouquereau, E., Lahiani, F. J., Beltou, N., Gimenes, G., & Gillet, N. (2016). Examining the Longitudinal Effects of Workload on Ill-being Through Each Dimension of Workaholism. *International Journal of Stress Management*, *25*, 144-162.

111 Ibrahim, S., Smith, P., & Muntaner, C. (2009). A multi-group cross-lagged analyses of work stressors and health using Canadian National sample. *Social Science & Medicine*, *68*, 49-59.

112 In-Albon, T., Meyer, A. H., Metzke, C. W., & Steinhausen, H. C. (2017). A Cross-Lag Panel Analysis of Low Self-Esteem as a Predictor of Adolescent Internalizing Symptoms in a Prospective Longitudinal Study. *Child Psychiatry & Human Development*, *48*, 411-422.

113 Jackson, S. L., & Cunningham, S. A. (2017). The stability of children's weight status over time, and the role of television, physical activity, and diet. *Preventive Medicine*, *100*, 229-234.

114 Jäggi, L., Drazdowski, T. K., & Kliewer, W. (2016). What parents don't know: disclosure and secrecy in a sample of urban adolescents. *Journal of Adolescence*, *53*, 64-74.

115 Jansen, P. W., Giallo, R., Westrupp, E. M., Wake, M., & Nicholson, J. M. (2013). Bidirectional associations between mothers' and fathers' parenting consistency and child BMI. *Pediatrics*, *132*, e1513-e1520.

116 Kashdan, T. B., Adams, L. M., Farmer, A. S., Ferssizidis, P., McKnight, P. E., & Nezlek, J. B. (2014). Sexual healing: Daily diary investigation of the benefits of intimate and pleasurable sexual activity in socially anxious adults. *Archives of Sexual Behavior*, *43*, 1417-1429.

117 Keijsers, L., Branje, S., Hawk, S. T., Schwartz, S. J., Frijns, T., Koot, H. M., van Lier, P., & Meeus, W. (2012). Forbidden friends as forbidden fruit: Parental supervision of friendships, contact with deviant peers, and adolescent delinquency. *Child Development*, *83*, 651-666.

118 Keles, S., Idsøe, T., Friborg, O., Sirin, S., & Oppedal, B. (2017). The longitudinal relation between daily hassles and depressive symptoms among unaccompanied refugees in Norway. *Journal of Abnormal Child Psychology*, *45*, 1413-1427.

119 Kilian, R., Lauber, C., Kalkan, R., Dorn, W., Rössler, W., Wiersma, D., ... & Becker, T. (2012). The relationships between employment, clinical status, and psychiatric hospitalisation in patients with schizophrenia receiving either IPS or a conventional vocational rehabilitation programme. *Social Psychiatry and Psychiatric Epidemiology*, *47*, 1381-1389.

120 Kim, H., Duran, C. A., Cameron, C. E., & Grissmer, D. (2018). Developmental relations among motor and cognitive processes and mathematics skills. *Child Development*, *89*, 476-494.

121 Kimonis, E. R., Centifanti, L. C., Allen, J. L., & Frick, P. J. (2014). Reciprocal Influences between Negative Life Events and Callous-Unemotional Traits. *Journal of Abnormal Child Psychology*, *42*, 1287-1298.

122 Kiviruusu, O., Berg, N., Huurre, T., Aro, H., Marttunen, M., & Haukkala, A. (2016). Interpersonal conflicts and development of self-esteem from adolescence to mid-adulthood. A 26-year follow-up. *PLoS ONE*, *11*, e0164942.

123 Klaas, H. S., Clémence, A., Marion-Veyron, R., Antonietti, J. P., Alameda, L., Golay, P., & Conus, P. (2017). Insight as a social identity process in the evolution of psychosocial functioning in the early phase of psychosis. *Psychological Medicine*, *47*, 718-729.

124 Klimstra, T. A., Luyckx, K., Hale III, W. W., & Goossens, L. (2014). Personality and externalizing behavior in the transition to young adulthood: the additive value of personality facets. *Social Psychiatry and Psychiatric Epidemiology*, *49*, 1319-1333.

125 Kochel, K. P., Ladd, G. W., & Rudolph, K. D. (2012). Longitudinal associations among youth depressive symptoms, peer victimization, and low peer acceptance: An interpersonal process perspective. *Child Development*, *83*, 637-650.

127 Koleck, M., Gana, K., Lucot, C., Darrigrand, B., Mazaux, J. M., & Glize, B. (2017). Quality of life in aphasic patients 1 year after a first stroke. *Quality of Life Research*, *26*, 45-54.

128 Konttinen, H., Kiviruusu, O., Huurre, T., Haukkala, A., Aro, H., & Marttunen, M. (2014). Longitudinal associations between depressive symptoms and body mass index in a 20-year follow-up. *International Journal of Obesity*, *38*, 668-674.

129 Kuijpers, R. C., Kleinjan, M., Engels, R. C., Stone, L. L., & Otten, R. (2015). Child self-report to identify internalizing and externalizing problems and the influence of maternal mental health. *Journal of Child and Family Studies*, *24*, 1605-1614.

130 Kuja-Halkola, R., Lichtenstein, P., D'Onofrio, B. M., & Larsson, H. (2015). Codevelopment of ADHD and externalizing behavior from childhood to adulthood. *Journal of Child Psychology and Psychiatry*, *56*, 640-647.

131 Labhart, F., Kuntsche, E., Wicki, M., & Gmel, G. (2017). Reciprocal influences of drinking motives on alcohol use and related consequences: A full cross-lagged panel study among young adult men. *Behavioral Medicine*, *43*, 277-284.

132 Lange, A. M., van der Rijken, R. E., Delsing, M. J., Busschbach, J. J., van Horn, J. E., & Scholte, R. H. (2017). Alliance and adherence in a systemic therapy. *Child and Adolescent Mental Health*, *22*, 148-154.

133 Lanz, M. A. R. G. H. E. R. I. T. A., & Tagliabue, S. (2014). Supportive relationships within ongoing families: Cross-lagged effects between components of support and adjustment in parents and young adult children. *Journal of Adolescence*, *37*, 1489-1503.

134 Lavigne, J. V., Hopkins, J., Gouze, K. R., & Bryant, F. B. (2015). Bidirectional influences of anxiety and depression in young children. *Journal of Abnormal Child Psychology*, *43*, 163-176.

135 Leadbeater, B. J., & Homel, J. (2015). Irritable and defiant sub-dimensions of ODD: Their stability and prediction of internalizing symptoms and conduct problems from adolescence to young adulthood. *Journal of Abnormal Child Psychology*, *43*, 407-421.

136 Leadbeater, B. J., & Hoglund, W. L. (2009). The effects of peer victimization and physical aggression on changes in internalizing from first to third grade. *Child Development*, *80*, 843-859.

137 Lewis, G., Collishaw, S., Thapar, A., & Harold, G. (2014). Parent–child hostility and child and adolescent depression symptoms: The direction of effects, role of genetic factors and gender. *European Child & Adolescent Psychiatry*, *23*, 317-327.

138 Li, T., & Zhang, Y. (2015). Social network types and the health of older adults: Exploring reciprocal associations. *Social Science & Medicine*, *130*, 59-68.

139 Tikotzky, L., & Sadeh, A. (2009). Maternal sleep-related cognitions and infant sleep: A longitudinal study from pregnancy through the 1st Year. *Child Development*, *80*, 860-874.

140 Lifshitz-Vahav, H., Shrira, A., & Bodner, E. (2017). The reciprocal relationship between participation in leisure activities and cognitive functioning: the moderating effect of self-rated literacy level. *Aging & Mental Health*, *21*, 524-531.

141 Lindwall, M., Larsman, P., & Hagger, M. S. (2011). The reciprocal relationship between physical activity and depression in older European adults: A prospective cross-lagged panel design using SHARE data. *Health Psychology*, *30*, 453-462.

142 Liu, H., Waite, L. J., Shen, S., & Wang, D. H. (2016). Is sex good for your health? A national study on partnered sexuality and cardiovascular risk among older men and women. *Journal of Health and Social Behavior*, *57*, 276-296.

143 Loukas, A. (2009). Examining temporal associations between perceived maternal psychological control and early adolescent internalizing problems. *Journal of Abnormal Child Psychology*, *37*, 1113-1122.

144 Lowe, S. R., Walsh, K., Uddin, M., Galea, S., & Koenen, K. C. (2014). Bidirectional relationships between trauma exposure and posttraumatic stress: a longitudinal study of Detroit residents. *Journal of Abnormal Psychology*, *123*, 533-544.

Riglin, L., Frederickson, N., Shelton, K. H., & Rice, F. (2013). A longitudinal study of
145 psychological functioning and academic attainment at the transition to secondary school. *Journal of Adolescence*, *36*, 507-517.

Luengo Kanacri, B. P., Eisenberg, N., Thartori, E., Pastorelli, C., Uribe Tirado, L. M., Gerbino,
146 M., & Caprara, G. V. (2017). Longitudinal relations among positivity, perceived positive school climate, and prosocial behavior in Colombian adolescents. *Child Development*, *88*, 1100-1114.

147 Luo, Y., Hawkley, L. C., Waite, L. J., & Cacioppo, J. T. (2012). Loneliness, health, and mortality in old age: A national longitudinal study. *Social Science & Medicine*, *74*, 907-914.

Luyckx, K., Missotten, L., Goossens, E., Moons, P., & i-Detach Investigators (2012). Individual
148 and contextual determinants of quality of life in adolescents with congenital heart disease. *Journal of Adolescent Health*, *51*, 122-128.

Luyckx, K., Seiffge-Krenke, I., & Hampson, S. E. (2010). Glycemic control, coping, and
149 internalizing and externalizing symptoms in adolescents with type 1 diabetes: a cross-lagged longitudinal approach. *Diabetes Care*, *33*, 1424-1429.

150 Magee, C., Caputi, P., & Iverson, D. (2014). Lack of sleep could increase obesity in children and too much television could be partly to blame. *Acta Pædiatrica*, *103*, e27-e31.

Mannering, A. M., Harold, G. T., Leve, L. D., Shelton, K. H., Shaw, D. S., Conger, R. D.,
151 Neiderhiser, J. M., Scaramella, L. V., & Reiss, D (2011). Longitudinal associations between marital instability and child sleep problems across infancy and toddlerhood in adoptive families. *Child Development*, *82*, 1252-1266.

Marschall-Lévesque, S., Castellanos-Ryan, N., Parent, S., Renaud, J., Vitaro, F., Boivin, M.,
152 Tremblay, R. E., & Séguin, J. R. (2017). Victimization, suicidal ideation, and alcohol use from age 13 to 15 years: Support for the self-medication model. *Journal of Adolescent Health*, *60*, 380-387.

153 Marshall, S. L., Parker, P. D., Ciarrochi, J., & Heaven, P. C. (2014). Is self‐esteem a cause or consequence of social support? A 4‐year longitudinal study. *Child Development*, *85*, 1275-1291.

Marsiglio, M. C., Chronister, K. M., Gibson, B., & Leve, L. D. (2014). Examining the link
154 between traumatic events and delinquency among juvenile delinquent girls: A longitudinal study. *Journal of Child & Adolescent Trauma*, *7*, 217-225.

Martinent, G., & Nicolas, M. (2017). Temporal ordering of affective states and coping within a
155 naturalistic achievement-related demanding situation. *International Journal of Stress Management*, *24*, 29-51.

Martz, M. E., Trucco, E. M., Cope, L. M., Hardee, J. E., Jester, J. M., Zucker, R. A., & Heitzeg,
156 M. M. (2016). Association of marijuana use with blunted nucleus accumbens response to reward anticipation. *JAMA Psychiatry*, *73*, 838-844.

Masquillier, C., Wouters, E., Mortelmans, D., & le Roux Booysen, F. (2015). The impact of
157 community support initiatives on the stigma experienced by people living with HIV/AIDS in
South Africa. *AIDS and Behavior*, *19*, 214-226.

Mauno, S., Feldt, T., Tolvanen, A., Hyvönen, K., & Kinnunen, U. (2011). Prospective
158 relationships between career disruptions and subjective well-being: evidence from a three-wave
follow-up study among Finnish managers. *International Archives of Occupational and Environmental Health*, *84*, 501-512.

McAdams, T. A., Salekin, R. T., Marti, C. N., Lester, W. S., & Barker, E. D. (2014). Co-
159 occurrence of antisocial behavior and substance use: Testing for sex differences in the impact of
older male friends, low parental knowledge and friends' delinquency. *Journal of Adolescence*, *37*, 247-256.

McAdams, T. A., Rijsdijk, F. V., Neiderhiser, J. M., Narusyte, J., Shaw, D. S., Natsuaki, M. N., ...
160 & Eley, T. C. (2015). The relationship between parental depressive symptoms and offspring
psychopathology: evidence from a children-of-twins study and an adoption study. *Psychological Medicine*, *45*, 2583-2594.

Meier, L. L., Tschudi, P., Meier, C. A., Dvorak, C., & Zeller, A. (2015). When general
161 practitioners don't feel appreciated by their patients: prospective effects on well-being and work–
family conflict in a Swiss Longitudinal Study. *Family Practice*, *32*, 181-186.

Micalizzi, L., Ronald, A., & Saudino, K. J. (2016). A genetically informed cross-lagged analysis
162 of autistic-like traits and affective problems in early childhood. *Journal of Abnormal Child Psychology*, *44*, 937-947.

Miller, A. B., Jenness, J. L., Oppenheimer, C. W., Gottlieb, A. L. B., Young, J. F., & Hankin, B.
163 L. (2017). Childhood Emotional Maltreatment as a Robust Predictor of Suicidal Ideation: A 3-
Year Multi-Wave, Prospective Investigation. *Journal of Abnormal Child Psychology*, *45*, 105-116.

Miller, A. L., Kaciroti, N., Sturza, J., Retzloff, L., Rosenblum, K., Vazquez, D. M., & Lumeng, J.
164 C. (2017). Associations between stress biology indicators and overweight across toddlerhood.
Psychoneuroendocrinology, *79*, 98-106.

Mitchison, D., Morin, A., Mond, J., Slewa-Younan, S., & Hay, P. (2015). The bidirectional
165 relationship between quality of life and eating disorder symptoms: A 9-year community-based
study of Australian women. *PLoS ONE*, *10*, e0120591.

Moberg, T., Lichtenstein, P., Forsman, M., & Larsson, H. (2011). Internalizing behavior in
166 adolescent girls affects parental emotional overinvolvement: A cross-lagged twin study. *Behavior Genetics*, *41*, 223-233.

Mrug, S., & Windle, M. (2009). Bidirectional influences of violence exposure and adjustment in
167 early adolescence: Externalizing behaviors and school connectedness. *Journal of Abnormal Child Psychology*, *37*, 611-623.

Muratori, P., Lochman, J. E., Lai, E., Milone, A., Nocentini, A., Pisano, S., Righini, E., & Masi,
168 G. (2016). Which dimension of parenting predicts the change of callous unemotional traits in
children with disruptive behavior disorder?. *Comprehensive Psychiatry*, *69*, 202-210.

169 Murphy, S., Elklit, A., Murphy, J., Hyland, P., & Shevlin, M. (2017). A cross-lagged panel study of dissociation and posttraumatic stress in a treatment-seeking sample of survivors of childhood sexual abuse. *Journal of Clinical Psychology*, *73*, 1370-1381.

170 Mustillo, S. A., Hendrix, K. L., & Schafer, M. H. (2012). Trajectories of body mass and self-concept in black and white girls: the lingering effects of stigma. *Journal of Health and Social Behavior*, *53*, 2-16.

171 Natsuaki, M. N., Leve, L. D., Harold, G. T., Neiderhiser, J. M., Shaw, D. S., Ganiban, J., Scaramella, L. V., & Reiss, D. (2013). Transactions between Child Social Wariness and Observed Structured Parenting: Evidence from a Prospective Adoption Study. *Child Development*, *84*, 1750-65.

172 Neece, C. L., Green, S. A., & Baker, B. L. (2012). Parenting stress and child behavior problems: A transactional relationship across time. *American Journal on Intellectual and Developmental Disabilities*, *117*, 48-66.

173 Negriff, S., Brensilver, M., & Trickett, P. K. (2015). Elucidating the mechanisms linking early pubertal timing, sexual activity, and substance use for maltreated versus nonmaltreated adolescents. *Journal of Adolescent Health*, *56*, 625-631.

174 Newland, R. P., Ciciolla, L., & Crnic, K. A. (2015). Crossover effects among parental hostility and parent–child relationships during the preschool period. *Journal of Child and Family Studies*, *24*, 2107-2119.

175 Nielsen, M. B., Birkeland, M. S., Hansen, M. B., Knardahl, S., & Heir, T. (2017). Victimization from workplace bullying after a traumatic event: time-lagged relationships with symptoms of posttraumatic stress. *International Archives of Occupational and Environmental Health*, *90*, 411-421.

176 Nishiguchi, Y., Takano, K., & Tanno, Y. (2016). The need for cognition mediates and moderates the association between depressive symptoms and impaired effortful control. *Psychiatry Research*, *241*, 8-13.

177 Occhipinti, S., Chambers, S. K., Lepore, S., Aitken, J., & Dunn, J. (2015). A longitudinal study of post-traumatic growth and psychological distress in colorectal cancer survivors. *PLoS ONE*, *10*, e0139119.

178 Olesen, S. C., Butterworth, P., Leach, L. S., Kelaher, M., & Pirkis, J. (2013). Mental health affects future employment as job loss affects mental health: findings from a longitudinal population study. *BMC Psychiatry*, *13*, 144.

179 Paek, M. S., Ip, E. H., Levine, B., & Avis, N. E. (2016). Longitudinal reciprocal relationships between quality of life and coping strategies among women with breast cancer. *Annals of Behavioral Medicine*, *50*, 775-783.

180 Palosaari, E., Punamäki, R. L., Diab, M., & Qouta, S. (2013). Posttraumatic cognitions and posttraumatic stress symptoms among war-affected children: A cross-lagged analysis. *Journal of Abnormal Psychology*, *122*, 656-661.

Palosaari, E., Punamäki, R. L., Peltonen, K., Diab, M., & Qouta, S. R. (2016). Negative social
181 relationships predict posttraumatic stress symptoms among war-affected children via
posttraumatic cognitions. *Journal of Abnormal Child Psychology*, *44*, 845-857.

Pastorelli, C., Lansford, J. E., Luengo Kanacri, B. P., Malone, P. S., Di Giunta, L., Bacchini, D.,
182 ... & Tapanya, S. (2016). Positive parenting and children's prosocial behavior in eight countries.
Journal of Child Psychology and Psychiatry, *57*, 824-834.

Patalay, P., Sharpe, H., & Wolpert, M. (2015). Internalising symptoms and body dissatisfaction:
183 untangling temporal precedence using cross-lagged models in two cohorts. *Journal of Child
Psychology and Psychiatry*, *56*, 1223-1230.

Pearl, A. M., French, B. F., Dumas, J. E., Moreland, A. D., & Prinz, R. (2014). Bidirectional
184 effects of parenting quality and child externalizing behavior in predominantly single parent,
under-resourced African American families. *Journal of Child and Family Studies*, *23*, 177-188.

Peter, R., March, S., & du Prel, J. B. (2016). Are status inconsistency, work stress and work-
185 family conflict associated with depressive symptoms? Testing prospective evidence in the lidA
study. *Social Science & Medicine*, *151*, 100-109.

186 Pettersson, C., Özdemir, M., & Eriksson, C. (2011). Effects of a parental program for preventing
underage drinking-The NGO program strong and clear. *BMC Public Health*, *11*, 251.

Peyre, H., Galera, C., Van Der Waerden, J., Hoertel, N., Bernard, J. Y., Melchior, M., & Ramus,
187 F. (2016). Relationship between early language skills and the development of
inattention/hyperactivity symptoms during the preschool period: Results of the EDEN mother-
child cohort. *BMC Psychiatry*, *16*, 380.

Pickard, H., Rijsdijk, F., Happé, F., & Mandy, W. (2017). Are social and communication
188 difficulties a risk factor for the development of social anxiety?. *Journal of the American Academy
of Child & Adolescent Psychiatry*, *56*, 344-351.

Poirier, M., Déry, M., Temcheff, C. E., Toupin, J., Verlaan, P., & Lemelin, J. P. (2016).
189 Longitudinal associations between conduct problems and depressive symptoms among girls and
boys with early conduct problems. *European Child & Adolescent Psychiatry*, *25*, 743-754.

Pössel, P., & Black, S. W. (2014). Testing three different sequential mediational interpretations of
190 Beck's cognitive model of the development of depression. *Journal of Clinical Psychology*, *70*,
72-94.

Preckel, F., Niepel, C., Schneider, M., & Brunner, M. (2013). Self-concept in adolescence: A
191 longitudinal study on reciprocal effects of self-perceptions in academic and social domains.
Journal of Adolescence, *36*, 1165-1175.

Priest, N., Perry, R., Ferdinand, A., Kelaher, M., & Paradies, Y. (2017). Effects over time of self-
192 reported direct and vicarious racial discrimination on depressive symptoms and loneliness among
Australian school students. *BMC Psychiatry*, *17*, 50.

193 Rapee, R. M. (2009). Early Adolescents' Perceptions of Their Mother's Anxious Parenting as a Predictor of Anxiety Symptoms 12 Months Later. *Journal of Abnormal Child Psychology* , *37* , 1103-1112.

194 Rawal, A., Riglin, L., Ng‐Knight, T., Collishaw, S., Thapar, A., & Rice, F. (2014). A longitudinal high‐risk study of adolescent anxiety, depression and parent‐severity on the developmental course of risk‐adjustment. *Journal of Child Psychology and Psychiatry* , *55* , 1270-1278.

195 Rhodes, R. E., Spence, J. C., Berry, T., Deshpande, S., Faulkner, G., Latimer-Cheung, A. E., O'Reilly, N., & Tremblay, M. S. (2015). Predicting changes across 12 months in three types of parental support behaviors and mothers’ perceptions of child physical activity. *Annals of Behavioral Medicine* , *49* , 853-864.

196 Ribeiro, L. A., Zachrisson, H. D., Schjolberg, S., Aase, H., Rohrer-Baumgartner, N., & Magnus, P. (2011). Attention problems and language development in preterm low-birth-weight children: Cross-lagged relations from 18 to 36 months. *BMC Pediatrics* , *11* , 59.

197 Richardson, M., Katsakou, C., & Priebe, S. (2011). Association of treatment satisfaction and psychopathological sub-syndromes among involuntary patients with psychotic disorders. *Social Psychiatry and Psychiatric Epidemiology* , *46* , 695-702.

198 Ritchie, S. J., Bates, T. C., & Plomin, R. (2015). Does learning to read improve intelligence? A longitudinal multivariate analysis in identical twins from age 7 to 16. *Child Development* , *86* , 23-36.

199 Richter, A., Schraml, K., & Leineweber, C. (2015). Work–family conflict, emotional exhaustion and performance-based self-esteem: reciprocal relationships. *International Archives of Occupational and Environmental Health* , *88* , 103-112.

200 Rivas‐Drake, D., Umaña‐Taylor, A. J., Schaefer, D. R., & Medina, M. (2017). Ethnic‐racial identity and friendships in early adolescence. *Child Development* , *88* , 710-724.

201 Rommel, A. S., Rijsdijk, F., Greven, C. U., Asherson, P., & Kuntsi, J. (2015). A longitudinal twin study of the direction of effects between ADHD symptoms and IQ. *PLoS ONE* , *10* , e0124357.

202 Ruttle, P. L., Maslowsky, J., Armstrong, J. M., Burk, L. R., & Essex, M. J. (2015). Longitudinal associations between diurnal cortisol slope and alcohol use across adolescence: A seven-year prospective study. *Psychoneuroendocrinology* , *56* , 23-28.

203 Salihovic, S., Kerr, M., Özdemir, M., & Pakalniskiene, V. (2012). Directions of effects between adolescent psychopathic traits and parental behavior. *Journal of Abnormal Child Psychology* , *40* , 957-969.

204 Savage, J., Verhulst, B., Copeland, W., Althoff, R. R., Lichtenstein, P., & Roberson-Nay, R. (2015). A genetically informed study of the longitudinal relation between irritability and anxious/depressed symptoms. *Journal of the American Academy of Child & Adolescent Psychiatry* , *54* , 377-384.

Sentse, M., Prinzie, P., & Salmivalli, C. (2017). Testing the Direction of Longitudinal Paths
205 between Victimization, Peer Rejection, and Different Types of Internalizing Problems in
Adolescence. *Journal of Abnormal Child Psychology*, *45*, 1013-1023.

Seymour, K. E., Chronis-Tuscano, A., Iwamoto, D. K., Kurdziel, G., & MacPherson, L. (2014).
206 Emotion regulation mediates the association between ADHD and depressive symptoms in a
community sample of youth. *Journal of Abnormal Child Psychology*, *42*, 611-621.

Shaffer, A., Lindhiem, O., Kolko, D. J., & Trentacosta, C. J. (2013). Bidirectional relations
207 between parenting practices and child externalizing behavior: A cross-lagged panel analysis in the
context of a psychosocial treatment and 3-year follow-up. *Journal of Abnormal Child
Psychology*, *41*, 199-210.

Shields, R. T., & Beaver, K. M. (2011). The effects of nonshared environments on adolescent
208 depression: Findings from a sample of monozygotic twins. *Journal of Adolescent Health*, *48*,
572-578.

209 Shimazu, A., & de Jonge, J. (2009). Reciprocal relations between effort–reward imbalance at
work and adverse health: A three-wave panel survey. *Social Science & Medicine*, *68*, 60-68.

Skalická, V., Belsky, J., Stenseng, F., & Wichstrøm, L. (2015). Reciprocal Relations Between
210 Student–Teacher Relationship and Children's Behavioral Problems: Moderation by Child-Care
Group Size. *Child Development*, *86*, 1557-1570.

Solberg, Ø., Birkeland, M. S., Blix, I., Hansen, M. B., & Heir, T. (2016). Towards an exposure-
211 dependent model of post-traumatic stress: longitudinal course of post-traumatic stress
symptomatology and functional impairment after the 2011 Oslo bombing. *Psychological
Medicine*, *46*, 3241-3254.

Song, T. M., An, J. Y., Hayman, L. L., Kim, G. S., Lee, J. Y., & Jang, H. L. (2012). A three-year
212 autoregressive cross-lagged panel analysis on nicotine dependence and average smoking.
Healthcare Informatics Research, *18*, 115-124.

Spanos, A., Klump, K. L., Burt, S. A., McGue, M., & Iacono, W. G. (2010). A longitudinal
213 investigation of the relationship between disordered eating attitudes and behaviors and parent–
child conflict: A monozygotic twin differences design. *Journal of Abnormal Psychology*, *119*,
293-299.

Spilt, J. L., Van Lier, P. A., Leflot, G., Onghena, P., & Colpin, H. (2014). Children's social self-
214 concept and internalizing problems: The influence of peers and teachers. *Child Development*, *85*,
1248-1256.

Stavrakakis, N., de Jonge, P., Ormel, J., & Oldehinkel, A. J. (2012). Bidirectional prospective
215 associations between physical activity and depressive symptoms. The TRAILS Study. *Journal of
Adolescent Health*, *50*, 503-508.

Stinglhamber, F., Marique, G., Caesens, G., Desmette, D., Hansez, I., Hanin, D., & Bertrand, F.
216 (2015). Employees' organizational identification and affective organizational commitment: An
integrative approach. *PLoS ONE*, *10*, e0123955.

217 Stratton, K. J., Clark, S. L., Hawn, S. E., Amstadter, A. B., Cifu, D. X., & Walker, W. C. (2014). Longitudinal interactions of pain and posttraumatic stress disorder symptoms in US Military service members following blast exposure. *The Journal of Pain*, *15*, 1023-1032.

218 Sturaro, C., Van Lier, P. A., Cuijpers, P., & Koot, H. M. (2011). The role of peer relationships in the development of early school-age externalizing problems. *Child Development*, *82*, 758-765.

219 Sutin, A. R., & Zonderman, A. B. (2012). Depressive symptoms are associated with weight gain among women. *Psychological Medicine*, *42*, 2351-2360.

220 Szabó, D., Kökönyei, G., Arató, A., Dezsofi, A., Molnár, K., Müller, K. E., ... & Veres, G. (2014). Autoregressive cross-lagged models of IMPACT-III and Pediatric Crohn's Disease Activity indexes during one year infliximab therapy in pediatric patients with Crohn's disease. *Journal of Crohn's and Colitis*, *8*, 747-755.

221 Tabri, N., Murray, H. B., Thomas, J. J., Franko, D. L., Herzog, D. B., & Eddy, K. T. (2015). Overvaluation of body shape/weight and engagement in non-compensatory weight-control behaviors in eating disorders: is there a reciprocal relationship?. *Psychological Medicine*, *45*, 2951-2958.

222 Tang, D., & Wang, D. (2009). Reason or result? Subjective well-being of the elderly in urban Beijing: A cross-lagged panel regression analysis. *Ageing International*, *34*, 189-202.

223 Taylor, M. J., Charman, T., Robinson, E. B., Plomin, R., Happé, F., Asherson, P., & Ronald, A. (2013). Developmental associations between traits of autism spectrum disorder and attention deficit hyperactivity disorder: a genetically informative, longitudinal twin study. *Psychological Medicine*, *43*, 1735-1746.

224 Taylor, J. L., Smith, L. E., & Mailick, M. R. (2014). Engagement in vocational activities promotes behavioral development for adults with autism spectrum disorders. *Journal of Autism and Developmental Disorders*, *44*, 1447-1460.

225 Talley, A. E., Aranda, F., Hughes, T. L., Everett, B., & Johnson, T. P. (2015). Longitudinal associations among discordant sexual orientation dimensions and hazardous drinking in a cohort of sexual minority women. *Journal of Health and Social Behavior*, *56*, 225-245.

226 Teppers, E., Luyckx, K., Klimstra, T. A., & Goossens, L. (2014). Loneliness and Facebook motives in adolescence: A longitudinal inquiry into directionality of effect. *Journal of Adolescence*, *37*, 691-699.

227 Tiet, Q. Q., Huizinga, D., & Byrnes, H. F. (2010). Predictors of resilience among inner city youths. *Journal of Child and Family Studies*, *19*, 360-378.

228 Tiggelman, D., van de Ven, M. O., van Schayck, O. C., & Engels, R. C. (2015). Longitudinal associations between asthma control, medication adherence, and quality of life among adolescents: results from a cross-lagged analysis. *Quality of Life Research*, *24*, 2067-2074.

229 Timmermans, M., van Lier, P. A., & Koot, H. M. (2010). The role of stressful events in the development of behavioural and emotional problems from early childhood to late adolescence. *Psychological Medicine* , *40* , 1659-1668.

230 Ma, T. L., & Bellmore, A. (2012). Peer victimization and parental psychological control in adolescence. *Journal of Abnormal Child Psychology* , *40* , 413-424.

231 Trucco, E. M., Villafuerte, S., Heitzeg, M. M., Burmeister, M., & Zucker, R. A. (2014). Rule breaking mediates the developmental association between GABRA 2 and adolescent substance abuse. *Journal of Child Psychology and Psychiatry* , *55* , 1372-1379.

232 Tsai, W., Nguyen, D. J., Weiss, B., Ngo, V., & Lau, A. S. (2017). Cultural differences in the reciprocal relations between emotion suppression coping, depressive symptoms and interpersonal functioning among adolescents. *Journal of Abnormal Child Psychology* , *45* , 657-669.

233 Tseng, W. L., Kawabata, Y., Gau, S. S. F., & Crick, N. R. (2014). Symptoms of Attention-Deficit/Hyperactivity Disorder and Peer Functioning: a Transactional Model of Development. *Journal of Abnormal Child Psychology* , *42* , 1353-1365.

234 Tucker, J. S., Miles, J. N., & D'Amico, E. J. (2013). Cross-lagged associations between substance use-related media exposure and alcohol use during middle school. *Journal of Adolescent Health* , *53* , 460-464.

235 Tucker, J. S., Miles, J. N., D'Amico, E. J., Zhou, A. J., Green Jr, H. D., & Shih, R. A. (2013). Temporal associations of popularity and alcohol use among middle school students. *Journal of Adolescent Healt* , *52* , 108-115.

236 Usami, S., Hayes, T., & McArdle, J. J. (2015). On the mathematical relationship between latent change score and autoregressive cross-lagged factor approaches: Cautions for inferring causal relationship between variables. *Multivariate Behavioral Research* , *50* , 676-687.

237 Van Dorn, R. A., Grimm, K. J., Desmarais, S. L., Tueller, S. J., Johnson, K. L., & Swartz, M. S. (2017). Leading indicators of community-based violent events among adults with mental illness. *Psychological Medicine* , *47* , 1179-1191.

238 Van Dulmen, M. H., Klipfel, K. M., Mata, A. D., Schinka, K. C., Claxton, S. E., Swahn, M. H., & Bossarte, R. M. (2012). Cross-lagged effects between intimate partner violence victimization and suicidality from adolescence into adulthood. *Journal of Adolescent Health* , *51* , 510-516.

239 Van Zalk, N., & Tillfors, M. (2017). Co-rumination buffers the link between social anxiety and depressive symptoms in early adolescence. *Child and Adolescent Psychiatry and Mental Health* , *11* , 41.

240 Vanhalst, J., Luyckx, K., Scholte, R. H., Engels, R. C., & Goossens, L. (2013). Low self-esteem as a risk factor for loneliness in adolescence: Perceived-but not actual-social acceptance as an underlying mechanism. *Journal of Abnormal Child Psychology* , *41* , 1067-1081.

241 Vaz, S., Falkmer, M., Parsons, R., Passmore, A. E., Parkin, T., & Falkmer, T. (2014). School belongingness and mental health functioning across the primary-secondary transition in a mainstream sample: Multi-group cross-lagged analyses. *PLoS ONE* , *9* , e99576.

242 Vella, S. A., Swann, C., Allen, M. S., Schweickle, M. J., & Magee, C. A. (2017). Bidirectional Associations between Sport Involvement and Mental Health in Adolescence. *Medicine and Science in Sports and Exercise* , *49* , 687-694.

243 Vitezova, A., Voortman, T., Zillikens, M. C., Jansen, P. W., Hofman, A., Uitterlinden, A. G., Franco, O. H., & Kiefte-de Jong, J. C. (2015). Bidirectional associations between circulating vitamin D and cholesterol levels: The Rotterdam Study. *Maturitas* , *82* , 411-417.

244 von Salisch, M., Denham, S. A., & Koch, T. (2017). Emotion knowledge and attention problems in young children: A cross-lagged panel study on the direction of effects. *Journal of Abnormal Child Psychology* , *45* , 45-56.

245 Von Stumm, S., & Deary, I. J. (2013). Intellect and cognitive performance in the Lothian Birth Cohort 1936. *Psychology and Aging* , *28* , 680-684.

246 Voss, P., Wolff, J. K., & Rothermund, K. (2017). Relations between views on ageing and perceived age discrimination: A domain-specific perspective. *European Journal of Ageing* , *14* , 5-15.

247 Waller, R., Gardner, F., Viding, E., Shaw, D. S., Dishion, T. J., Wilson, M. N., & Hyde, L. W. (2014). Bidirectional associations between parental warmth, callous unemotional behavior, and behavior problems in high-risk preschoolers. *Journal of Abnormal Child Psychology* , *42* , 1275-1285.

248 Wang, M. T., & Fredricks, J. A. (2014). The reciprocal links between school engagement, youth problem behaviors, and school dropout during adolescence. *Child Development* , *85* , 722-737.

249 Wang, M. T., & Kenny, S. (2014). Parental physical punishment and adolescent adjustment: Bidirectionality and the moderation effects of child ethnicity and parental warmth. *Journal of Abnormal Child Psychology* , *42* , 717-730.

250 Wang, M. T., & Kenny, S. (2014). Longitudinal links between fathers’ and mothers’ harsh verbal discipline and adolescents’ conduct problems and depressive symptoms. *Child Development* , *85* , 908-923.

251 Wang, M. V., Lekhal, R., Aarø, L. E., & Schjølberg, S. (2014). Co-occurring development of early childhood communication and motor skills: results from a population-based longitudinal study. *Child: care, health and development* , *40* , 77-84.

252 Webb, E., Panico, L., Bécares, L., McMunn, A., Kelly, Y., & Sacker, A. (2017). The inter-relationship of adolescent unhappiness and parental mental distress. *Journal of Adolescent Health* , *60* , 196-203.

253 Weinstein, N., Przybylski, A. K., & Murayama, K. (2017). A prospective study of the motivational and health dynamics of Internet Gaming Disorder. *PeerJ* , *5* , e3838.

254 Welp, A., Meier, L. L., & Manser, T. (2016). The interplay between teamwork, clinicians' emotional exhaustion, and clinician-rated patient safety: a longitudinal study. *Critical Care* , *20* , 110.

255 Whelan, Y. M., Leibenluft, E., Stringaris, A., & Barker, E. D. (2015). Pathways from maternal depressive symptoms to adolescent depressive symptoms: the unique contribution of irritability symptoms. *Journal of Child Psychology and Psychiatry* , *56* , 1092-1100.

256 Wichstrøm, L., & von Soest, T. (2016). Reciprocal relations between body satisfaction and self-esteem: A large 13-year prospective study of adolescents. *Journal of Adolescence* , *47* , 16-27.

257 Wickrama, K. A. S., Hwa Kwag, K., Lorenz, F. O., Conger, R. D., & Surjadi, F. F. (2010). Dynamics of family economic hardship and the progression of health problems of husbands and wives during the middle years: A perspective from rural Mid-West. *Journal of Aging and Health* , *22* , 1132-1157.

258 Williams, L. R., & Steinberg, L. (2011). Reciprocal relations between parenting and adjustment in a sample of juvenile offenders. *Child Development* , *82* , 633-645.

259 Wolf, E. J., Bovin, M. J., Green, J. D., Mitchell, K. S., Stoop, T. B., Barretto, K. M., ... & Marx, B. P. (2016). Longitudinal associations between post-traumatic stress disorder and metabolic syndrome severity. *Psychological Medicine* , *46* , 2215-2226.

260 Wolff, U. (2011). Effects of a randomised reading intervention study: An application of structural equation modelling. *Dyslexia* , *17* , 295-311.

261 Wols, A., Scholte, R. H. J., & Qualter, P. (2015). Prospective associations between loneliness and emotional intelligence. *Journal of Adolescence* , *39* , 40-48.

262 Wood, J. J., Lynne-Landsman, S. D., Langer, D. A., Wood, P. A., Clark, S. L., Mark Eddy, J., & Ialongo, N. (2012). School Attendance Problems and Youth Psychopathology: Structural Cross-Lagged Regression Models in Three Longitudinal Data Sets. *Child Development* , *83* , 351-366.

263 Wouters, E., Masquillier, C., & le Roux Booysen, F. (2016). The Importance of the family: a longitudinal study of the predictors of depression in HIV patients in South Africa. *AIDS and Behavior* , *20* , 1591-1602.

264 Yan, N., & Dix, T. (2014). Mothers' early depressive symptoms and children's first-grade adjustment: A transactional analysis of child withdrawal as a mediator. *Journal of Child Psychology and Psychiatry* , *55* , 495-504.

Yu, G., Sessions, J. G., Fu, Y., & Wall, M. (2015). A multilevel cross-lagged structural equation
265 analysis for reciprocal relationship between social capital and health. *Social Science & Medicine*,
142, 1-8.

266 Zahl, T., Steinsbekk, S., & Wichstrøm, L. (2017). Physical activity, sedentary behavior, and symptoms of major depression in middle childhood. *Pediatrics*, *139*, e20161711.

Zavos, H. M., Rijsdijk, F. V., & Eley, T. C. (2012). A longitudinal, genetically informative, study
267 of associations between anxiety sensitivity, anxiety and depression. *Behavior Genetics*, *42*, 592-
602.

Zhou, X., Wu, X., An, Y., & Fu, F. (2014). Longitudinal relationships between posttraumatic
268 stress symptoms and sleep problems in adolescent survivors following the Wenchuan earthquake
in China. *PLoS ONE*, *9*, e104470.

Zhou, X., Wu, X., & Chen, J. (2015). Longitudinal linkages between posttraumatic stress disorder
269 and posttraumatic growth in adolescent survivors following the Wenchuan earthquake in China:
A three-wave, cross-lagged study. *Psychiatry Research*, *228*, 107-111.

270 Zhu, J., Yu, C., Bao, Z., Jiang, Y., Zhang, W., Chen, Y., ... & Zhang, J. (2017). Deviant peer affiliation as an explanatory mechanism in the association between corporal punishment and physical aggression: a longitudinal study among Chinese adolescents. *Journal of Abnormal Child Psychology*, *45*, 1537-1551.

van Den Eijnden, R. J., Spijkerman, R., Vermulst, A. A., van Rooij, T. J., & Engels, R. C. (2010).
271 Compulsive Internet use among adolescents: Bidirectional parent–child relationships. *Journal of*
Abnormal Child Psychology, *38*, 77-89.

Table B (a) Marginal means of estimates of cross-lagged parameters at each model

	Data generation model								
	CLPM			RI-CLPM			STARTS		
Analysis model	CLPM	RI-CLPM	STARTS	CLPM	RI-CLPM	STARTS	CLPM	RI-CLPM	STARTS
γ=0	0.00	0.00	0.04	0.00	0.00	0.04	0.00	0.00	0.02
γ=0.1	0.10	0.05	0.04	0.05	0.05	0.04	0.04	0.02	0.02
γ=0.2	0.21	0.10	0.05	0.10	0.11	0.03	0.09	0.04	0.02
β=0.5	0.10	0.06	0.05	0.05	0.06	0.04	0.04	0.02	0.02
β=0.7	0.11	0.04	0.04	0.06	0.04	0.03	0.05	0.01	0.01
ψ_2=0.2	0.10	0.04	0.05	0.05	0.05	0.05	0.05	0.03	0.03
ψ_2=0.4	0.10	0.05	0.04	0.05	0.05	0.03	0.05	0.02	0.01
ψ_2=0.6	0.10	0.05	0.04	0.06	0.06	0.02	0.04	0.01	0.01
N=200	0.10	0.05	0.05	0.05	0.05	0.04	0.05	0.02	0.02
N=600	0.10	0.05	0.04	0.05	0.05	0.03	0.05	0.02	0.01
N=1000	0.10	0.05	0.04	0.05	0.05	0.03	0.05	0.02	0.01
T=4	0.10	0.03	0.01	0.05	0.04	0.01	0.04	0.01	0.01
T=6	0.10	0.05	0.05	0.05	0.06	0.04	0.04	0.02	0.02
T=8	0.11	0.06	0.07	0.05	0.06	0.05	0.05	0.03	0.02

Table B (b) Marginal means of standardized estimates of cross-lagged parameters at each model

	Data generation model								
	CLPM			RI-CLPM			STARTS		
Analysis model	CLPM	RI-CLPM	STARTS	CLPM	RI-CLPM	STARTS	CLPM	RI-CLPM	STARTS
γ=0	0.00	0.00	0.08	0.00	0.00	0.38	0.00	0.00	0.08
γ=0.1	0.12	0.06	0.09	0.06	0.07	0.36	0.05	0.02	0.08
γ=0.2	0.24	0.12	0.18	0.11	0.14	0.38	0.10	0.05	0.10
β=0.5	0.13	0.08	0.08	0.05	0.09	0.25	0.04	0.03	0.08
β=0.7	0.11	0.05	0.15	0.06	0.05	0.57	0.06	0.01	0.09
ψ_2=0.2	0.13	0.06	0.11	0.05	0.07	0.25	0.05	0.03	0.21
ψ_2=0.4	0.12	0.06	0.13	0.06	0.07	0.38	0.05	0.02	0.04
ψ_2=0.6	0.11	0.06	0.11	0.06	0.06	0.48	0.04	0.01	0.01
N=200	0.12	0.06	0.11	0.06	0.07	0.34	0.05	0.02	0.09
N=600	0.12	0.06	0.12	0.06	0.07	0.38	0.05	0.02	0.09
N=1000	0.12	0.06	0.12	0.06	0.07	0.40	0.05	0.02	0.09
T=4	0.12	0.04	0.02	0.06	0.05	0.25	0.05	0.01	0.04
T=6	0.12	0.07	0.14	0.05	0.07	0.39	0.05	0.02	0.09
T=8	0.12	0.07	0.19	0.06	0.08	0.48	0.05	0.03	0.13

Table B (c) Marginal means of biases of estimates in cross-lagged parameters at each model

	Data generation model								
	CLPM			RI-CLPM			STARTS		
Analysis model	CLPM	RI-CLPM	STARTS	CLPM	RI-CLPM	STARTS	CLPM	RI-CLPM	STARTS
γ=0	0.00	0.00	0.04	0.00	0.00	0.04	0.00	0.00	0.02
γ=0.1	0.00	-0.05	-0.06	-0.05	-0.05	-0.06	-0.06	-0.08	-0.08
γ=0.2	0.01	-0.10	-0.15	-0.10	-0.09	-0.17	-0.11	-0.16	-0.18
β=0.5	0.00	-0.04	-0.05	-0.05	-0.04	-0.06	-0.06	-0.08	-0.08
β=0.7	0.01	-0.06	-0.06	-0.04	-0.06	-0.07	-0.05	-0.09	-0.09
ψ_2=0.2	0.00	-0.06	-0.05	-0.05	-0.05	-0.05	-0.05	-0.07	-0.07
ψ_2=0.4	0.00	-0.05	-0.06	-0.05	-0.05	-0.07	-0.05	-0.08	-0.09
ψ_2=0.6	0.00	-0.05	-0.06	-0.04	-0.04	-0.08	-0.06	-0.09	-0.09
N=200	0.00	-0.05	-0.05	-0.05	-0.05	-0.06	-0.05	-0.08	-0.08
N=600	0.00	-0.05	-0.06	-0.05	-0.05	-0.07	-0.05	-0.08	-0.09
N=1000	0.00	-0.05	-0.06	-0.05	-0.05	-0.07	-0.05	-0.08	-0.09
T=4	0.00	-0.07	-0.09	-0.05	-0.06	-0.09	-0.06	-0.09	-0.09
T=6	0.00	-0.05	-0.05	-0.05	-0.04	-0.06	-0.06	-0.08	-0.08
T=8	0.01	-0.04	-0.03	-0.05	-0.04	-0.05	-0.05	-0.07	-0.08
